# Supplementary material for: Decrypting the programming of β-methylation in virginiamycin M biosynthesis
Source: Nat Commun. 2023 Mar 10;14:1327. doi: 10.1038/s41467-023-36974-3 (PMC10006238; doi:10.1038/s41467-023-36974-3)
Supplement: Supplementary file 1 — Supplementary Information [file 41467_2023_36974_MOESM1_ESM.pdf]

Supplementary Information

Decrypting the programming of β-methylation in virginiamycin M biosynthesis

S. Collin, *et al.*

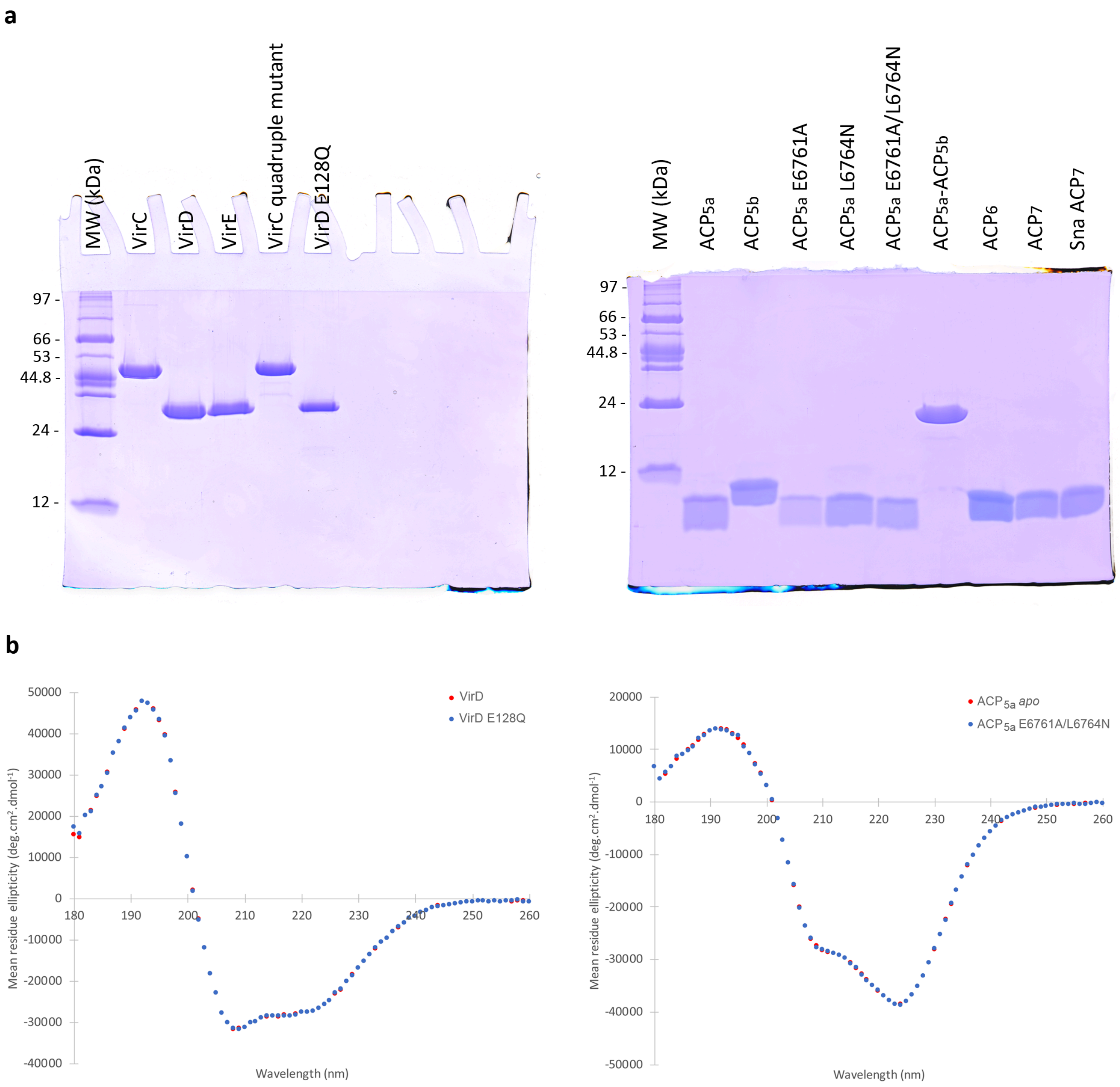

Supplementary Fig. 1 | Evidence for the purity of proteins and structural integrity of mutants used in this study. a. SDS-PAGE analysis of constructs used in this study. Gel 1: VirD (calc'd: 26.3 kDa); VirC (calc'd: 45.7 kDa); VirE (calc'd: 27.1 kDa); VirC quadruple mutant C114A/Q334A/R335A/R338A (calc'd: 45.5 kDa); VirD E128Q mutant (calc'd: 26.3 kDa). Gel 2: ACP$_{5a}$ (calc'd: 8.8 kDa); ACP$_{5b}$ (calc'd: 9.2 kDa); ACP$_{5a}$ E6761A (calc'd: 8.8 kDa); ACP$_{5a}$ L6764N (calc'd: 8.8 kDa); ACP$_{5a}$ E6761A/L6764N (calc'd: 8.8 kDa); ACP$_{5a}$-ACP$_{5b}$ (calc'd: 19.4 kDa); ACP$_{6}$ (calc'd: 8.8 kDa); ACP$_{7}$ (calc'd: 8.9 kDa); and, Sna ACP$_{7}$ (calc'd: 10.4 kDa). The molecular weights of the markers are indicated. **b.** CD analysis of select mutants. VirD vs. VirD E128Q (left) and ACP$_{5a}$ vs. ACP$_{5a}$ E6761A/L6764N (right). The data shown were obtained by averaging three data sets acquired consecutively on the same samples. As the spectra are essentially superimposable, this analysis showed that the mutations did not significantly alter the structure of each protein, although minor differences between ACP$_{5a}$ and the double mutant were detected. Source data are provided as a Source Data file. Abbreviations: Vir, virginiamycin; Sna, pristinamycin.

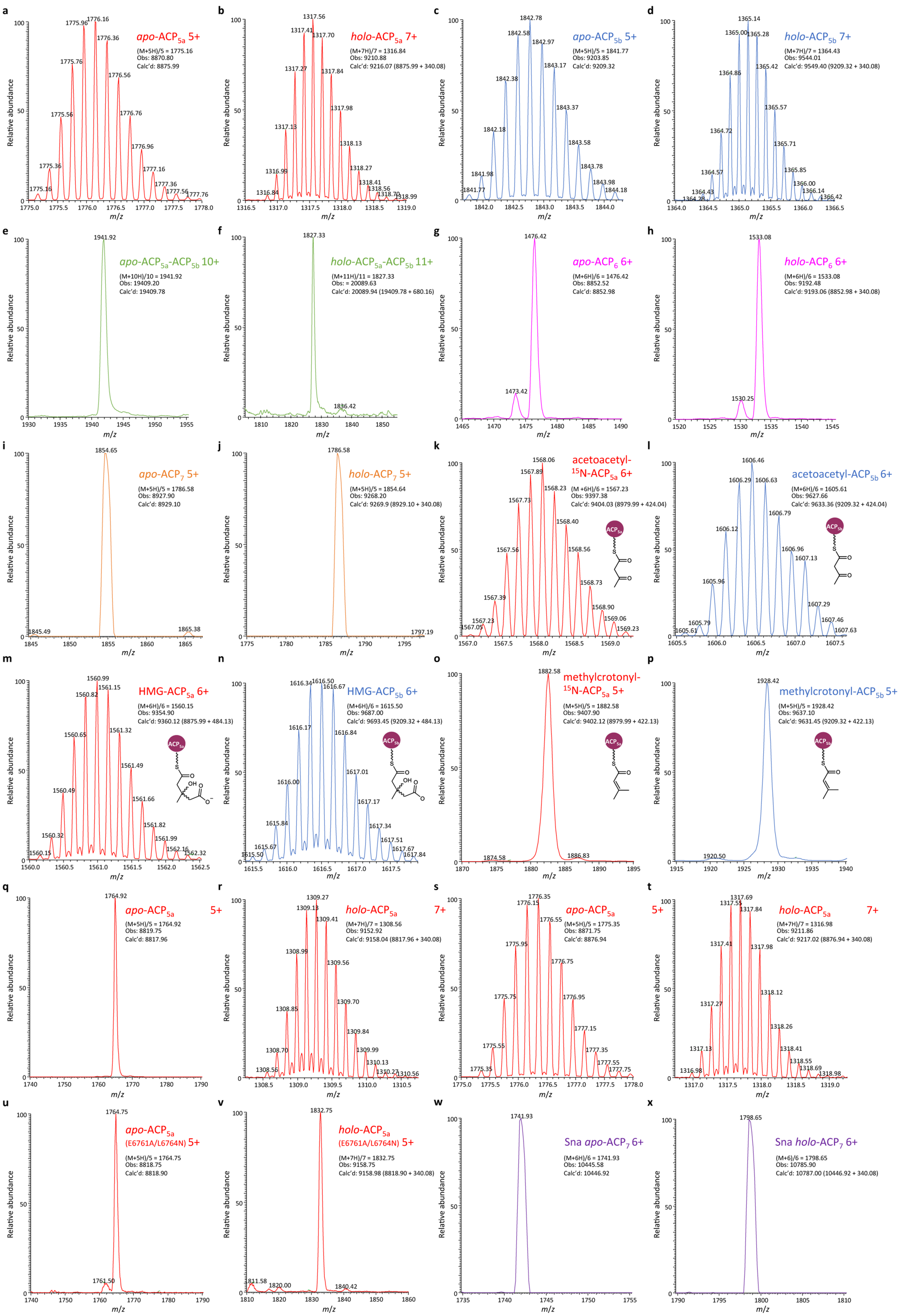
a
apo-ACP5a 5+
(M+5H)/5 = 1775.16
Obs: 8870.80
Calc'd: 8875.99
b
holo-ACP5a 7+
(M+7H)/7 = 1316.84
Obs: 9210.88
Calc'd: 9216.07 (8875.99 + 340.08)
c
apo-ACP5b 5+
(M+5H)/5 = 1841.77
Obs: 9203.85
Calc'd: 9209.32
d
holo-ACP5b 7+
(M+7H)/7 = 1364.43
Obs: 9544.01
Calc'd: 9549.40 (9209.32 + 340.08)
e
apo-ACP5a-ACP5b 10+
(M+10H)/10 = 1941.92
Obs: 19409.20
Calc'd: 19409.78
f
holo-ACP5a-ACP5b 11+
(M+11H)/11 = 1827.33
Obs: = 20089.63
Calc'd: 20089.94 (19409.78 + 680.16)
g
apo-ACP6 6+
(M+6H)/6 = 1476.42
Obs: 8852.52
Calc'd: 8852.98
h
holo-ACP6 6+
(M+6H)/6 = 1533.08
Obs: 9192.48
Calc'd: 9193.06 (8852.98 + 340.08)
i
apo-ACP7 5+
(M+5H)/5 = 1786.58
Obs: 8927.90
Calc'd: 8929.10
j
holo-ACP7 5+
(M+5H)/5 = 1854.64
Obs: 9268.20
Calc'd: 9269.9 (8929.10 + 340.08)
k
acetoacetyl-15N-ACP5a 6+
(M +6H)/6 = 1567.23
Obs: 9397.38
Calc'd: 9404.03 (8979.99 + 424.04)
l
acetoacetyl-ACP5b 6+
(M+6H)/6 = 1605.61
Obs: 9627.66
Calc'd: 9633.36 (9209.32 + 424.04)
m
HMG-ACP5a 6+
(M+6H)/6 = 1560.15
Obs: 9354.90
Calc'd: 9360.12 (8875.99 + 484.13)
n
HMG-ACP5b 6+
(M+6H)/6 = 1615.50
Obs: 9687.00
Calc'd: 9693.45 (9209.32 + 484.13)
o
methylcrotonyl-15N-ACP5a 5+
(M+5H)/5 = 1882.58
Obs: 9407.90
Calc'd: 9402.12 (8979.99 + 422.13)
p
methylcrotonyl-ACP5b 5+
(M+5H)/5 = 1928.42
Obs: 9637.10
Calc'd: 9631.45 (9209.32 + 422.13)
q
apo-ACP5a 5+
(M+5H)/5 = 1764.92
Obs: 8819.75
Calc'd: 8817.96
r
holo-ACP5a 7+
(M+7H)/7 = 1308.56
Obs: 9152.92
Calc'd: 9158.04 (8817.96 + 340.08)
s
apo-ACP5a 5+
(M+5H)/5 = 1775.35
Obs: 8871.75
Calc'd: 8876.94
t
holo-ACP5a 7+
(M+7H)/7 = 1316.98
Obs: 9211.86
Calc'd: 9217.02 (8876.94 + 340.08)
u
apo-ACP5a (E6761A/L6764N) 5+
(M+5H)/5 = 1764.75
Obs: 8819.75
Calc'd: 8818.90
v
holo-ACP5a (E6761A/L6764N) 5+
(M+7H)/7 = 1832.75
Obs: 9158.75
Calc'd: 9158.98 (8818.90 + 340.08)
w
Sna apo-ACP7 6+
(M+6H)/6 = 1741.93
Obs: 10445.58
Calc'd: 10446.92
x
Sna holo-ACP7 6+
(M+6H)/6 = 1798.65
Obs: 10785.90
Calc'd: 10787.00 (10446.92 + 340.08)
Relative abundance
m/z

Supplementary Fig. 2 | Verification of the modification states of the investigated ACP domains by HPLC-MS. The molecular weights of the following ACPs were determined by HPLC-MS (the ACPs were sourced from the virginiamycin system unless otherwise indicated): **a.** apo-ACP_{5a}; **b.** holo-ACP_{5a}; **c.** apo-ACP_{5b}; **d.** holo-ACP_{5b}; **e.** apo-ACP_{5a}-ACP_{5b}; **f.** holo-ACP_{5a}-ACP_{5b}; **g.** apo-ACP_{6}; **h.** holo-ACP_{6}; **i.** apo-ACP_{7}; **j.** holo-ACP_{7}; **k.** acetoacetyl-^{15}N-ACP_{5a}; **l.** acetoacetyl-ACP_{5b}; **m.** HMG-ACP_{5a}; **n.** HMG-ACP_{5b}; **o.** methylcrotonyl-^{15}N-ACP_{5a}; **p.** methylcrotonyl-ACP_{5b}; **q.** apo-ACP_{5a} (E6761A); **r.** holo-ACP_{5a} (E6761A); **s.** apo-ACP_{5a} (L6764N); **t.** holo-ACP_{5a} (L6764N); **u.** apo-ACP_{5a} (E6761A/L6764N); **v.** holo-ACP_{5a} (E6761A/L6764N); **w.** Sna apo-ACP_{7}; **x.** Sna holo-ACP_{7}. In each case, the data representing a single charge state are shown, along with the observed and the calculated molecular weights. For the analyses carried out at high-resolution (60K), the full suite of isotopic peaks is visible (in such cases the monoisotopic peak was used to calculate the observed molecular weight), while for analysis at lower resolution (3 or 7.5K), only a broad peak centred on the monoisotopic value was observed. For convenience, certain ^{15}N-labelled ACPs prepared for the NMR-based structure elucidation were modified and assayed, as indicated. In panels **k**–**p**, the structures of the three employed substrate analogues are shown. Abbreviations: ACP, acyl carrier protein; HMG-ACP, 3-hydroxy-3-methylglutaryl-ACP; Sna, pristinamycin.

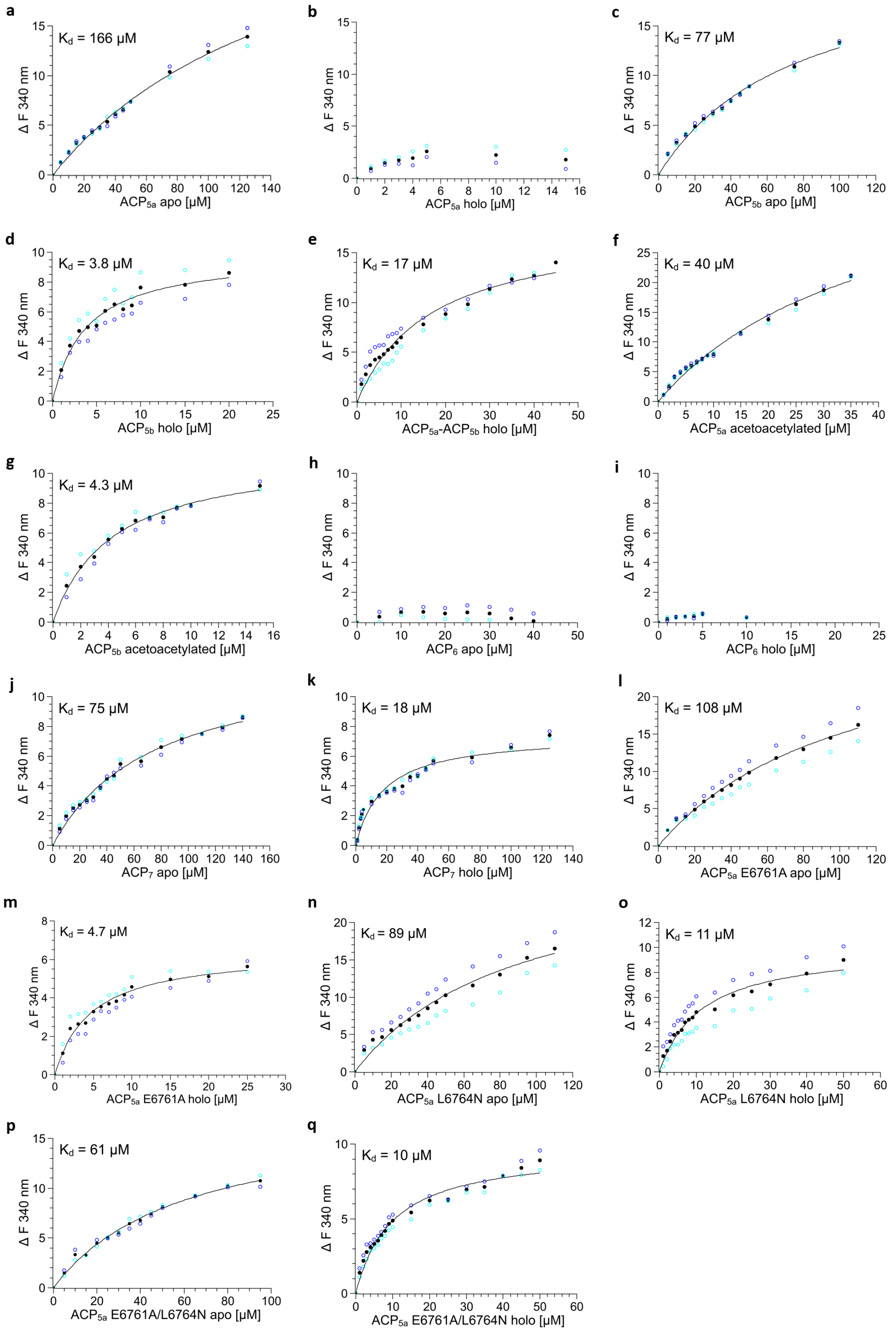
a
Kd = 166 µM
Δ F 340 nm
ACP5a apo [µM]
b
Δ F 340 nm
ACP5a holo [µM]
c
Kd = 77 µM
Δ F 340 nm
ACP5b apo [µM]
d
Kd = 3.8 µM
Δ F 340 nm
ACP5b holo [µM]
e
Kd = 17 µM
Δ F 340 nm
ACP5a-ACP5b holo [µM]
f
Kd = 40 µM
Δ F 340 nm
ACP5a acetoacetylated [µM]
g
Kd = 4.3 µM
Δ F 340 nm
ACP5b acetoacetylated [µM]
h
Δ F 340 nm
ACP6 apo [µM]
i
Δ F 340 nm
ACP6 holo [µM]
j
Kd = 75 µM
Δ F 340 nm
ACP7 apo [µM]
k
Kd = 18 µM
Δ F 340 nm
ACP7 holo [µM]
l
Kd = 108 µM
Δ F 340 nm
ACP5a E6761A apo [µM]
m
Kd = 4.7 µM
Δ F 340 nm
ACP5a E6761A holo [µM]
n
Kd = 89 µM
Δ F 340 nm
ACP5a L6764N apo [µM]
o
Kd = 11 µM
Δ F 340 nm
ACP5a L6764N holo [µM]
p
Kd = 61 µM
Δ F 340 nm
ACP5a E6761A/L6764N apo [µM]
q
Kd = 10 µM
Δ F 340 nm
ACP5a E6761A/L6764N holo [µM]

Supplementary Fig. 3 | Investigation of binding between VirC and ligand ACP domains by tryptophan fluorescence quenching. VirC (5 μM) fluorescence intensity in the presence of increasing concentrations of ligands was recorded by fluorescence spectroscopy. Ligand concentrations vs. changes in fluorescence intensity at 340 nm were then plotted for: **a.** apo-ACP_{5a}; **b.** holo-ACP_{5a}; **c.** apo-ACP_{5b}; **d.** holo-ACP_{5b}; **e.** holo-ACP_{5a}-ACP_{5b}; **f.** acetoacetyl-ACP_{5a}; **g.** acetoacetyl-ACP_{5b}; **h.** apo-ACP_{6}; **i.** holo-ACP_{6}; **j.** apo-ACP_{7}; **k.** holo-$ACP_{7;}$ **l.** apo-ACP_{5a} E6761Ap; **m.** holo-ACP_{5a} E6761A, **n.** apo-ACP_{5a} L6764N; **o.** holo-ACP_{5a} L6764N; **p.** apo-ACP_{5a} E6761A/L6764N apo; and, **q.** holo-ACP_{5a} E6761A/L6764N. The data obtained for each ACP concentration from two independent experiments are shown in blue and cyan hollow circles, and the calculated average values in solid black circles. The curve corresponding to the average values was analysed via nonlinear regression using a one site-specific binding model ($F = F_{max}*X/(K_d + X)$, where X is the ligand concentration, F is the fluorescence intensity, F_{max} is the maximum specific binding and K_d is the equilibrium binding constant) with SciDAVis v.2.3.0. The measured binding affinities are summarised in **Table 1**. Abbreviation: ACP, acyl carrier protein.

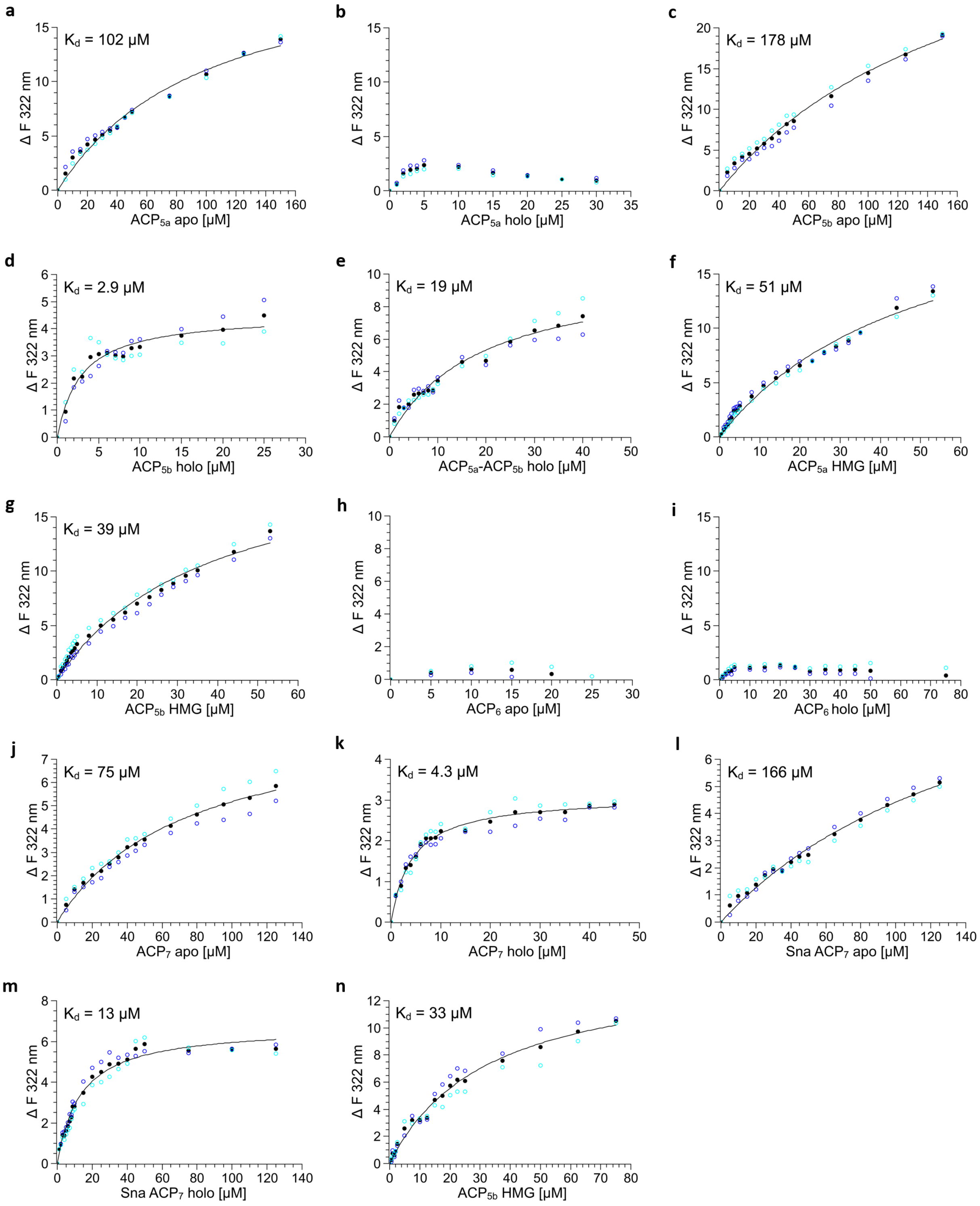
a
K$_d$ = 102 µM
Δ F 322 nm
ACP$_{5a}$ apo [µM]
b
Δ F 322 nm
ACP$_{5a}$ holo [µM]
c
K$_d$ = 178 µM
Δ F 322 nm
ACP$_{5b}$ apo [µM]
d
K$_d$ = 2.9 µM
Δ F 322 nm
ACP$_{5b}$ holo [µM]
e
K$_d$ = 19 µM
Δ F 322 nm
ACP$_{5a}$-ACP$_{5b}$ holo [µM]
f
K$_d$ = 51 µM
Δ F 322 nm
ACP$_{5a}$ HMG [µM]
g
K$_d$ = 39 µM
Δ F 322 nm
ACP$_{5b}$ HMG [µM]
h
Δ F 322 nm
ACP$_6$ apo [µM]
i
Δ F 322 nm
ACP$_6$ holo [µM]
j
K$_d$ = 75 µM
Δ F 322 nm
ACP$_7$ apo [µM]
k
K$_d$ = 4.3 µM
Δ F 322 nm
ACP$_7$ holo [µM]
l
K$_d$ = 166 µM
Δ F 322 nm
Sna ACP$_7$ apo [µM]
m
K$_d$ = 13 µM
Δ F 322 nm
Sna ACP$_7$ holo [µM]
n
K$_d$ = 33 µM
Δ F 322 nm
ACP$_{5b}$ HMG [µM]

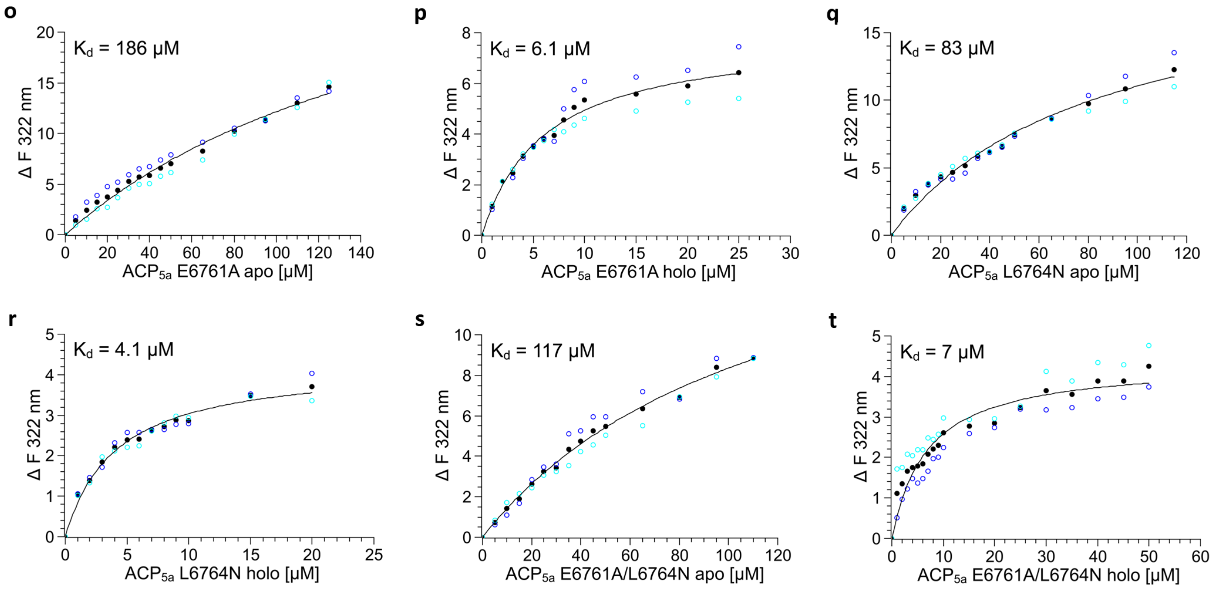

Supplementary Fig. 4 | Investigation of binding between VirD/VirD E128Q and ligand ACP domains by tryptophan fluorescence quenching. VirD or VirD E128Q (5 μM) fluorescence intensity in the presence of increasing concentrations of ligands was recorded by fluorescence spectroscopy. Ligand concentrations vs. changes in fluorescence intensity at 322 nm were plotted for: **a.** apo-ACP_{5a}; **b.** holo-ACP_{5a}; **c.** apo-ACP_{5b}; **d.** holo-ACP_{5b}; **e.** holo-ACP_{5a}-ACP_{5b}; **f.** HMG-ACP_{5a}; **g.** HMG-ACP_{5b}; **h.** apo-ACP_{6}; **i.** holo-ACP_{6}; **j.** apo-ACP_{7}; **k.** holo-ACP_{7}; **l.** Sna apo-ACP_{7}; **m.** Sna holo-ACP_{7}; **n.** VirD E128Q mutant and HMG-ACP_{5b}; **o.** apo-ACP_{5a} E6761A; **p.** holo-ACP_{5a} E6761A; **q.** apo-ACP_{5a} L6764N; **r.** holo-ACP_{5a} L6764N; **s.** apo-ACP_{5a} E6761A/L6764N; and, **t.** holo-ACP_{5a} E6761A/L6764N. The data obtained for each ACP concentration from two independent experiments are shown in blue and cyan hollow circles, and the calculated average values in solid black circles. The curve corresponding to the average values was analysed via nonlinear regression using a one site-specific binding model ($F = F_{max}*X/(K_d + X)$, where X is the ligand concentration, F is the fluorescence intensity, F_{max} is the maximum specific binding and K_d is the equilibrium binding constant) with SciDAVis v.2.3.0. The measured binding affinities are summarised in **Table 1**. Abbreviations: ACP, acyl carrier protein; HMG-ACP, 3-hydroxy-3-methylglutaryl-ACP; Sna, pristinamycin.

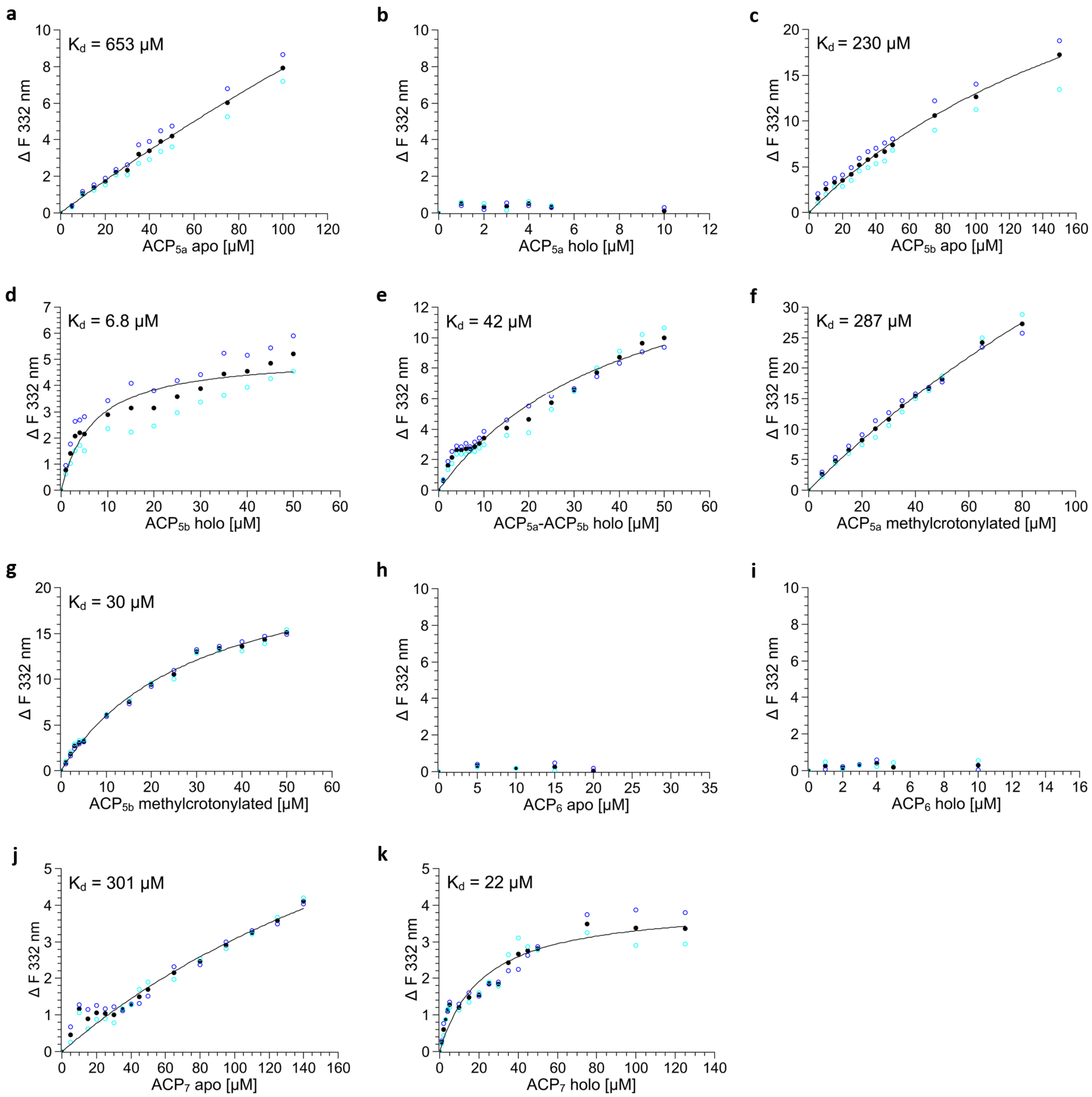

Supplementary Fig. 5 │ Investigation of binding between VirE and ligand ACP domains by tryptophan fluorescence quenching. VirE (5 µM) fluorescence intensity in the presence of increasing concentrations of ligands was recorded by fluorescence spectroscopy. Ligand concentrations vs. changes in fluorescence intensity at 332 nm were plotted for: **a.** apo-ACP_{5a}; **b.** holo-ACP_{5a}; **c.** apo-ACP_{5b}; **d.** holo-ACP_{5b}; **e.** holo-ACP_{5a}-ACP_{5b}; **f.** methylcrotonyl-ACP_{5a}; **g.** methylcrotonyl-ACP_{5b}; **h.** apo-ACP_6; **i.** holo-ACP_6; **j.** apo-ACP_7; and, **k.** holo-ACP_7. The data obtained for each ACP concentration from two independent experiments are shown in blue and cyan hollow circles, and the calculated average values in solid black circles. The curve corresponding to the average values was analysed via nonlinear regression using a one site-specific binding model ($F = F_{max}*X/(K_d + X)$, where X is the ligand concentration, F is the fluorescence intensity, F_{max} is the maximum specific binding and K_d is the equilibrium binding constant) with SciDAVis v.2.3.0. The measured binding affinities are summarised in **Table 1.** Abbreviation: ACP, acyl carrier protein.

a. holo-ACP$_{5b}$–VirC complex

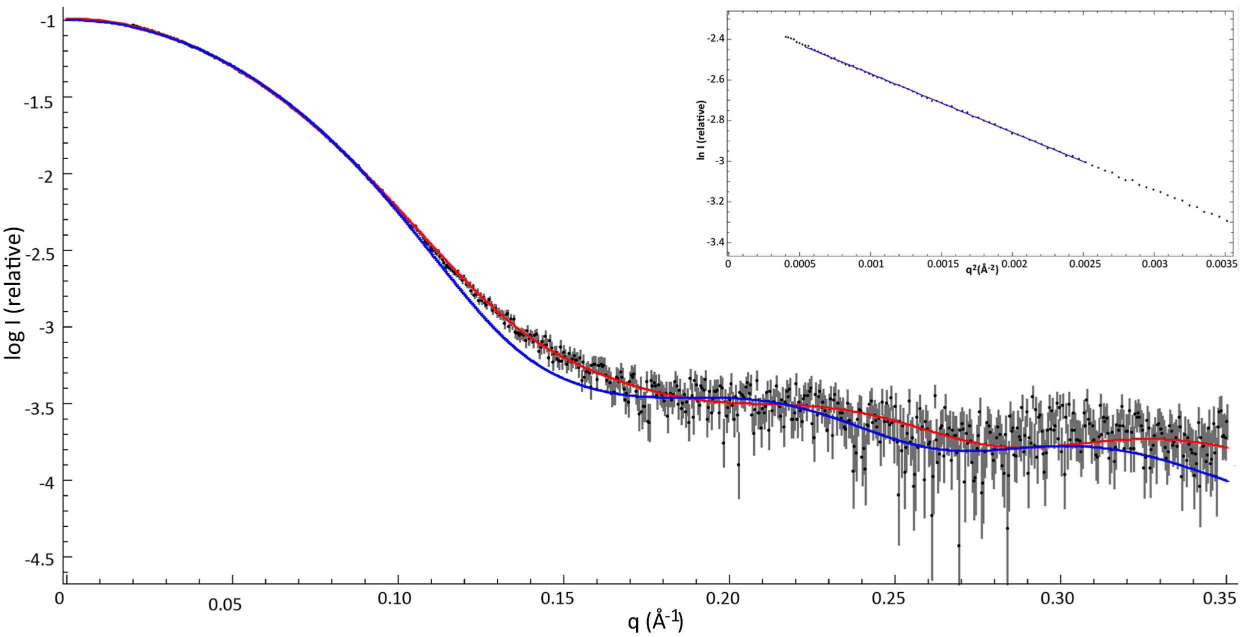

b. VirD

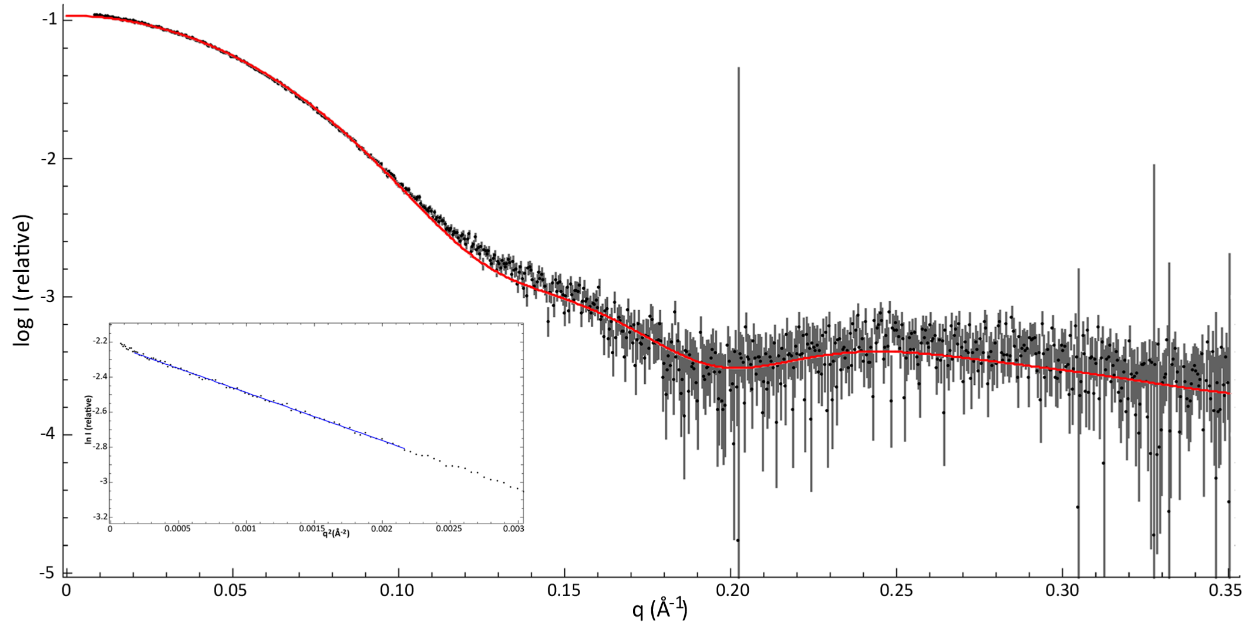

c. holo-ACP$_{5b}$–VirD complex

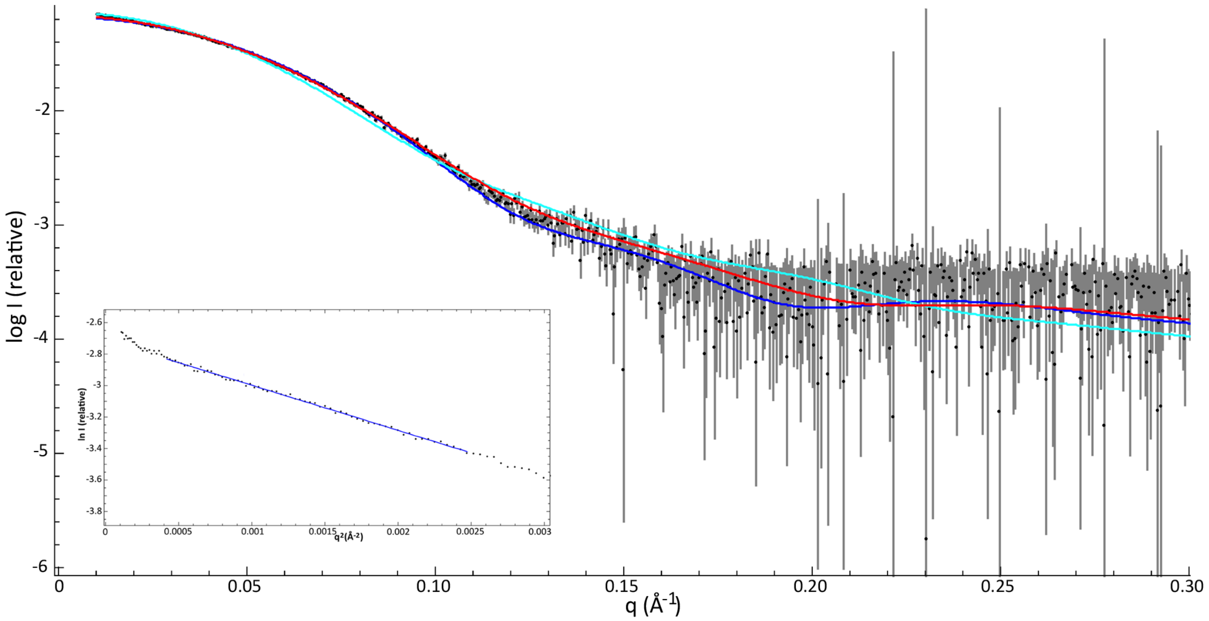

d. VirE

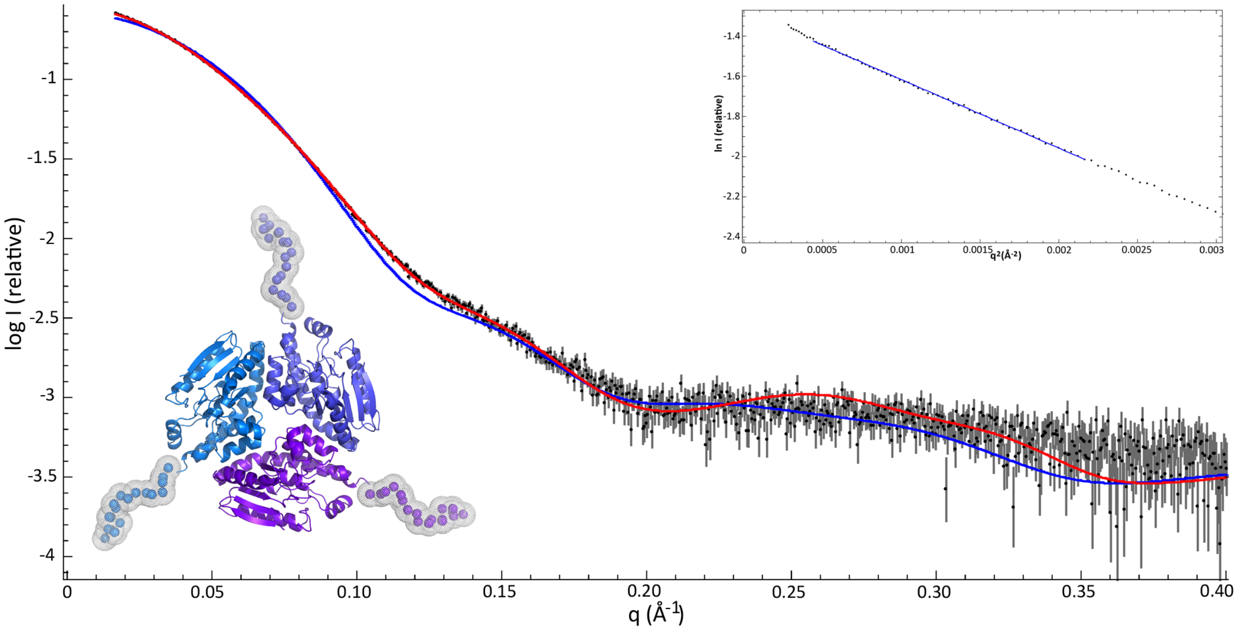

e. holo-ACP$_{5b}$–VirE complex

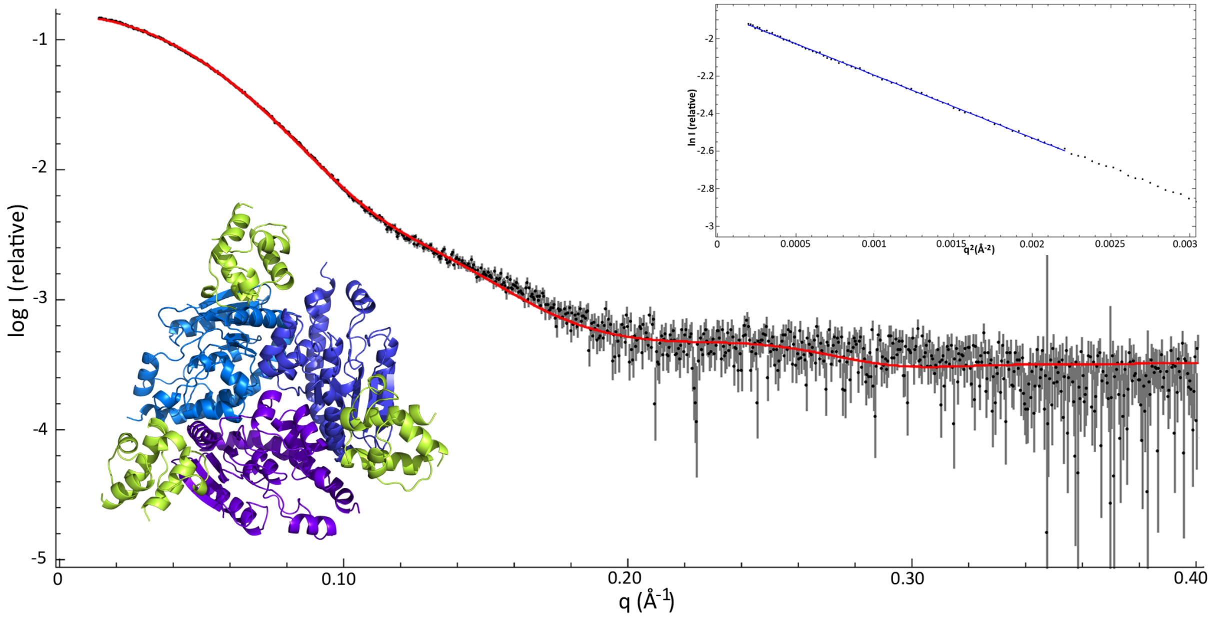

Supplementary Fig. 6 | Fits between experimental SAXS data and the crystal or modelled structures. a. Experimental SAXS curve obtained from the holo-ACP$_{5b}$–VirC complex (in black) fit to the theoretical scattering data calculated from the CurD dimer (in blue) and from the acetyl-ACP$_D$–CurD complex (PDB ID: 5KP6 [http://doi.org/10.2210/pdb5KP6/pdb]) (in red) using CRYSOL[1]. The Guinier plot is inset. The discrepancies between the theoretical scattering curves for dimeric CurD and the acetyl-ACP$_D$–CurD complex, and the experimentally determined data for the holo-ACP$_{5b}$–VirC complex (χ^2), were calculated at 6.19 and 1.52, respectively. **b.** Experimental SAXS curve obtained from VirD (in black) fit to the theoretical SAXS curve (χ^2 = 2.1) calculated from the crystal structure of VirD (PDB ID: 8AHZ [http://doi.org/10.2210/pdb8AHZ/pdb]) (in blue). The Guinier plot is inset. **c.** Experimental SAXS curve from the holo-ACP$_{5b}$–VirD complex (in black), with the Guinier plot inset. The fit to the theoretical SAXS curves generated from the crystal structure of holo-ACP$_{5b}$–VirD complex (PDB ID: 8AHQ [http://doi.org/10.2210/pdb8AHQ/pdb]) using CRYSOL[1] is shown in green (χ^2 = 10.3), and that to the crystal structure of VirD (PDB ID: 8AHZ [http://doi.org/10.2210/pdb8AHZ/pdb]) in blue (χ^2 = 2.10). The fit (in red) was subsequently improved (χ^2 = 1.72) by using OLIGOMER[2], which showed that a mixture of both VirD and holo-ACP$_{5b}$–VirD complex is present in solution, at a ratio of 7:3. Reasoning that the holo-ACP$_{5b}$–VirD complex was dissociating during the SEC-SAXS analysis, we attempted to force complex formation by increasing the ratio of holo-ACP$_{5b}$ to VirD (4:1 and 8:1). However, this failed to increase the proportion of obtained complex, reflecting one of the limits of the SAXS approach. **d.** Experimental SAXS curve obtained from VirE (in black), with the Guinier plot inset. Comparison to the theoretical SAXS curve (in cyan) derived from a model of trimeric VirE lacking the C-terminal helices α10 (last 18 residues) modelled by ColabFold[3], yielded a χ^2 of 18.1. We hypothesised that the unsatisfactory fits between the experimental and theoretical curves could reflect poorly-modelled monomer orientations within the trimer, as well as the positioning of loop regions and the flexible C-terminus. Indeed, a substantially improved fit (in red) to the data (χ^2 = 1.65) was obtained using CORAL[4] to model the trimer as well as the missing C-terminal helix α10, with imposition of three-fold symmetry. In the resulting SAXS model of trimeric VirE, the C-terminal 18 residues are extended, and the oligomeric arrangement is quite different from that predicted by ColabFold[3] (r.m.s.d. of 2.87 (688 Cα)). **e.** Experimental SAXS scattering curve obtained from the holo-ACP$_{5b}$–VirE complex (in black), with the Guinier plot inset. To interpret these data, we derived a model of the ACP$_{5b}$–VirE complex with SASREF[5], using versions of VirE and ACP$_{5b}$ lacking their unstructured regions (VirE helix α10 and the N- and C-termini of ACP$_{5b}$), in order improve the rigid-body modelling. Comparison of the resulting calculated SAXS curve[1] with the experimental data (in red) gave a good fit (χ^2 = 1.89). We attempted to improve the fit further by modelling the folded VirE helix α10 using the Ensemble Optimisation Method (EOM), but were unsuccessful. Indeed, multiple factors complicate an EOM-based analysis of this complex, including the fact that the VirE region which becomes ordered participates in the very interfaces that EOM is attempting to model by rigid-body docking, and the dominance of the SAXS signal by the larger VirE trimer. Nonetheless, the obtained data show clearly that the holo-ACP$_{5b}$-VirE complex resembles that of holo-ACP$_{5b}$-VirD, with the ACP$_{5b}$ domains positioned at the interfaces between two VirE monomers. Abbreviation: ACP, acyl carrier protein.

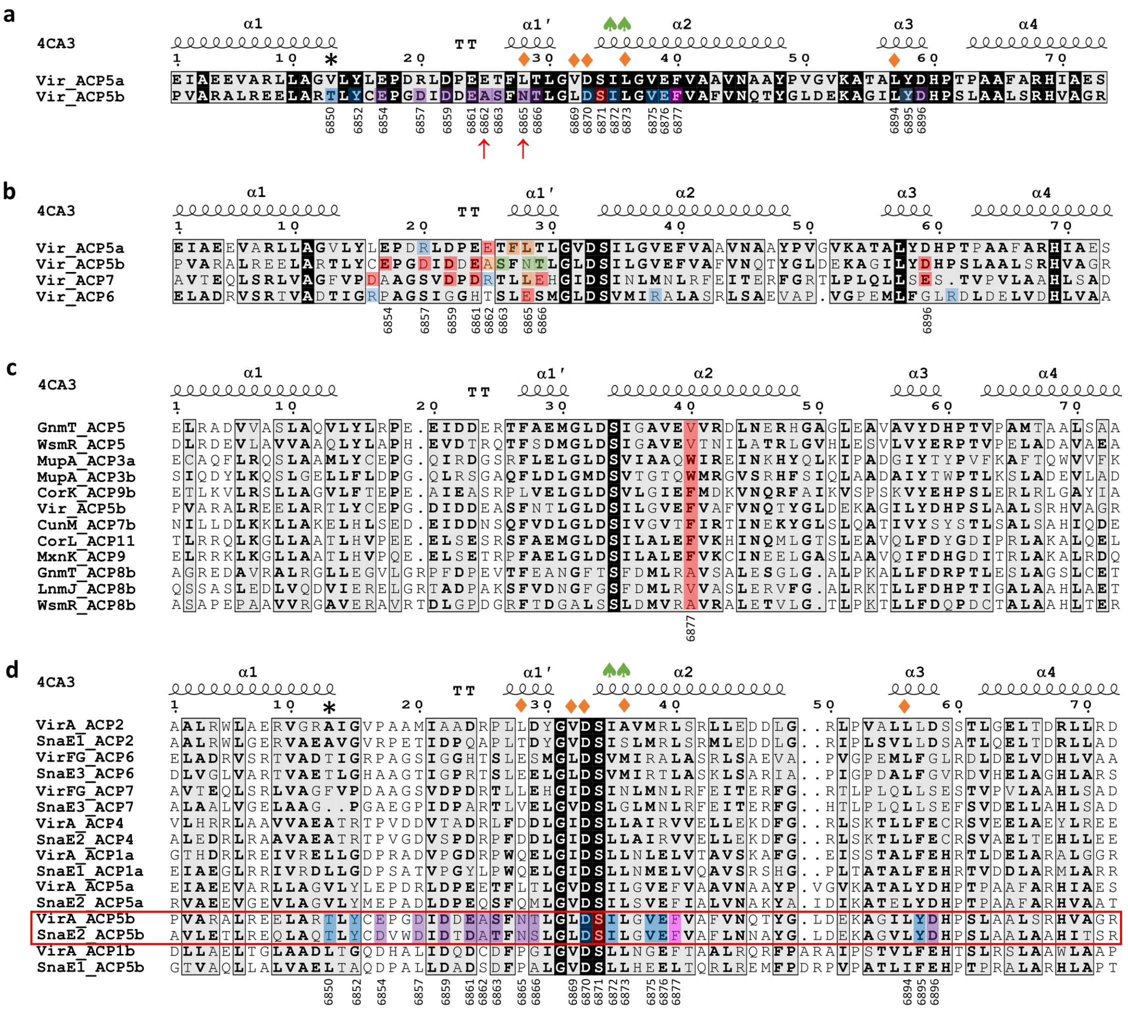

Supplementary Fig. 7 | Comparative sequence analysis of *trans*-AT PKS ACPs. a. Sequences of Vir ACP$_{5a}$ and ACP$_{5b}$. Legend: red = catalytic Ser; blue = residues contributing to the interface with VirD (*****, only residue position differing between ACP$_{5b}$ and ACP$_{5a}$); ♠ = residues comprising the Asp-binding oxyanion hole; ♦ = ACP/VirD specificity determinants, acting via a hydrogen-bond network; pink = position of the W flag; purple = residues contributing to key surface electrostatic features; ↑ = residues mutated in ACP$_{5a}$ to match those present in ACP$_{5b}$. Throughout the figure, the sequence numbering is based on VirA ACP$_{5b}$ (GenBank: BAF50727.1). The indicated secondary structure elements are those present in the NMR structure of Vir apo-ACP$_{5b}$ (PDB ID: 4CA3 [http://doi.org/10.2210/pdb4CA3/pdb][6]). **b.** Comparison between Vir ACP$_{5b}$ and ACP$_{7}$ which are recognised by the β-methylation cassette, and ACP$_{5a}$ and ACP$_{6}$ which are not, in terms of residues contributing to the respective electrostatic surface potentials (**Fig. 4**) (red indicates acidic resides, blue basic residues, orange hydrophobic amino acids, and green, polar residues). Sequence numbering is based on VirA ACP$_{5b}$[7]. **c.** Sequences of ACP domains from modules within select *trans*-AT PKSs in which β-modification occurs (the ACPs are numbered according to their modules of origin, i.e. ACP$_{5}$ = the fifth PKS module of the system, with the exception of MupA_ACP$_{3a}$/$_{3b}$, which are numbered according to ref. [7]). The labels 'a' and 'b' refer to the first and second domains, respectively, of tandem ACPs. Both MupA ACP$_{3a}$ and ACP$_{3b}$ are included as representative sequences upon which the W flag model was based[7] (residue position highlighted in red). Sequence numbering is based on VirA ACP$_{5b}$[7]. This analysis shows that like the Vir system, multiple β-modification ACPs incorporate an F at this position, but equally that other residues are present (V and A). Abbreviation: ACP, acyl carrier protein. Subunit names and accession numbers: Gnm, guangnanmycin[8] (GnmT, GenBank: ATY69569.1); Wsm, weishanmycin[8] (WsmR, GenBank: ATY69589.1); Mmp, mupirocin[7] (MmpA, GenBank: AAM12909.2); Cor, corallopyronin[9] (CorL, GenBank: ADI59534.1; CorK, GenBank: ADI59533.1); Cun, cuniculene[10] (CunM, GenBank: EZH71968.1); Mxn, myxopyronin[11] (MxnK, GenBank: AGS77291.1); Lnm, leinamycin[12] (LnmJ, GenBank: AAN85523.1). **d.** Multiple sequence alignment of all ACPs from the Vir and pristinamycin (Sna) systems. The key interaction positions in Vir ACP$_{5b}$, which are well conserved in SnaE2 ACP$_{5b}$, are highlighted. VirFG, ref. [6]. Accession numbers: SnaE1, GenBank: CBW45749.1; SnaE2, GenBank: CBW45748.1; SnaE3, GenBank: CBW45741.1.

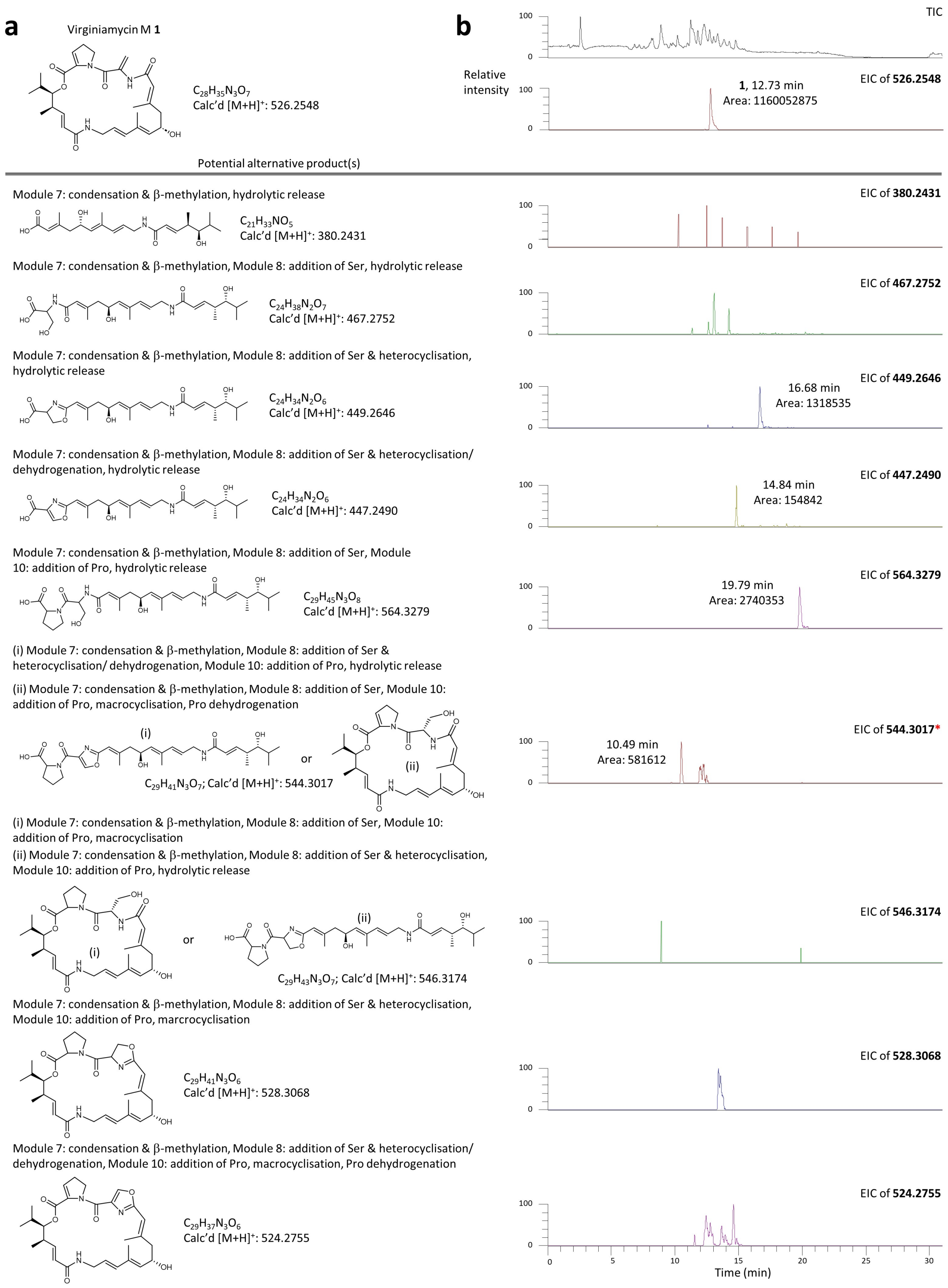
a
Virginiamycin M 1
C28H35N3O7
Calc'd [M+H]+: 526.2548
Potential alternative product(s)
Module 7: condensation & β-methylation, hydrolytic release
C21H33NO5
Calc'd [M+H]+: 380.2431
Module 7: condensation & β-methylation, Module 8: addition of Ser, hydrolytic release
C24H38N2O7
Calc'd [M+H]+: 467.2752
Module 7: condensation & β-methylation, Module 8: addition of Ser & heterocyclisation, hydrolytic release
C24H34N2O6
Calc'd [M+H]+: 449.2646
Module 7: condensation & β-methylation, Module 8: addition of Ser & heterocyclisation/ dehydrogenation, hydrolytic release
C24H34N2O6
Calc'd [M+H]+: 447.2490
Module 7: condensation & β-methylation, Module 8: addition of Ser, Module 10: addition of Pro, hydrolytic release
C29H45N3O8
Calc'd [M+H]+: 564.3279
(i) Module 7: condensation & β-methylation, Module 8: addition of Ser & heterocyclisation/ dehydrogenation, Module 10: addition of Pro, hydrolytic release
(ii) Module 7: condensation & β-methylation, Module 8: addition of Ser, Module 10: addition of Pro, macrocyclisation, Pro dehydrogenation
(i)
or
(ii)
C29H41N3O7; Calc'd [M+H]+: 544.3017
(i) Module 7: condensation & β-methylation, Module 8: addition of Ser, Module 10: addition of Pro, macrocyclisation
(ii) Module 7: condensation & β-methylation, Module 8: addition of Ser & heterocyclisation, Module 10: addition of Pro, hydrolytic release
(i)
or
(ii)
C29H43N3O7; Calc'd [M+H]+: 546.3174
Module 7: condensation & β-methylation, Module 8: addition of Ser & heterocyclisation, Module 10: addition of Pro, marcrocyclisation
C29H41N3O6
Calc'd [M+H]+: 528.3068
Module 7: condensation & β-methylation, Module 8: addition of Ser & heterocyclisation/ dehydrogenation, Module 10: addition of Pro, macrocyclisation, Pro dehydrogenation
C29H37N3O6
Calc'd [M+H]+: 524.2755
b
TIC
Relative intensity
EIC of 526.2548
1, 12.73 min
Area: 1160052875
EIC of 380.2431
EIC of 467.2752
EIC of 449.2646
16.68 min
Area: 1318535
EIC of 447.2490
14.84 min
Area: 154842
EIC of 564.3279
19.79 min
Area: 2740353
EIC of 544.3017*
10.49 min
Area: 581612
EIC of 546.3174
EIC of 528.3068
EIC of 524.2755
Time (min)

Supplementary Fig. 8 | High-resolution MS analysis for additional metabolites produced by wild type *S. pristinaespiralis* ATCC 25486. a. Theoretical products derived from β-methylation in module 7, followed by variable biosynthetic events catalysed by the domains located downstream, with carboxylic acids released by spontaneous hydrolysis. In each case, the metabolite structure, its molecular formula and calculated mass ($[M+H]^+$) are indicated. **b.** TIC of the extract, and EICs corresponding to the calculated masses in **a.** (mass tolerance set to 10 ppm), with the EIC of **1** shown as a control. For detected peaks, the retention times and measured peak areas are indicated. *The potential hydrated form of derivative **3**. Abbreviations: TIC, total ion chromatogram; EIC, extracted ion chromatogram.

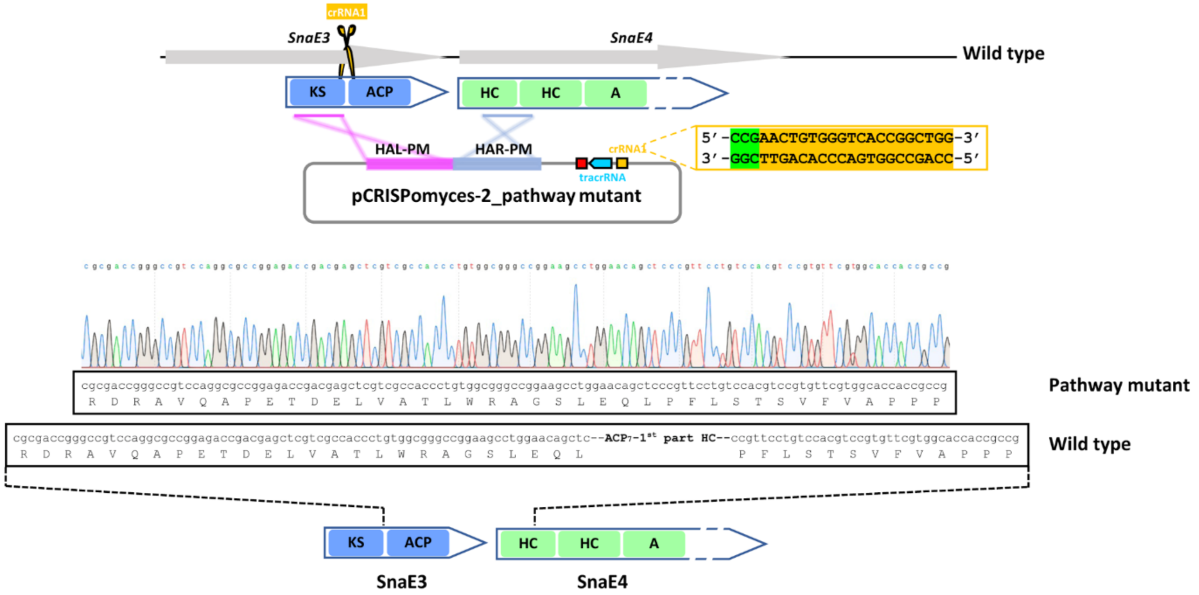

Supplementary Fig. 9 | Illustration of the strategy for generation of the Sna pathway inactivation mutant in *Streptomyces pristinaespiralis* ATCC 25486 by CRISPR-Cas9[13]. Schematic representation of pathway inactivation targeting the interface between modules 7 and 8. The sequencing confirming the identity of the targeted mutant is shown. Abbreviations: KS, ketosynthase; ACP, acyl carrier protein; HC, heterocyclisation; A, adenylation; HAL, homology arm left; HAR, homology arm right; PM, pathway mutant.

a.

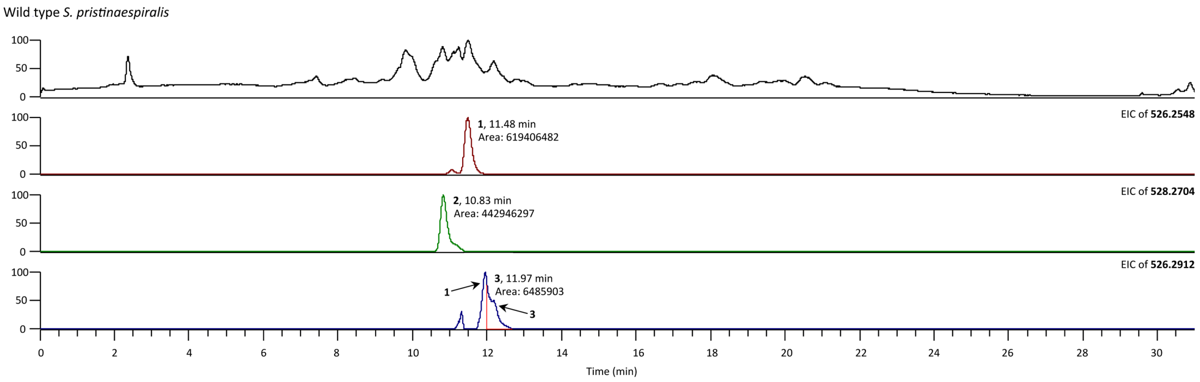

b.

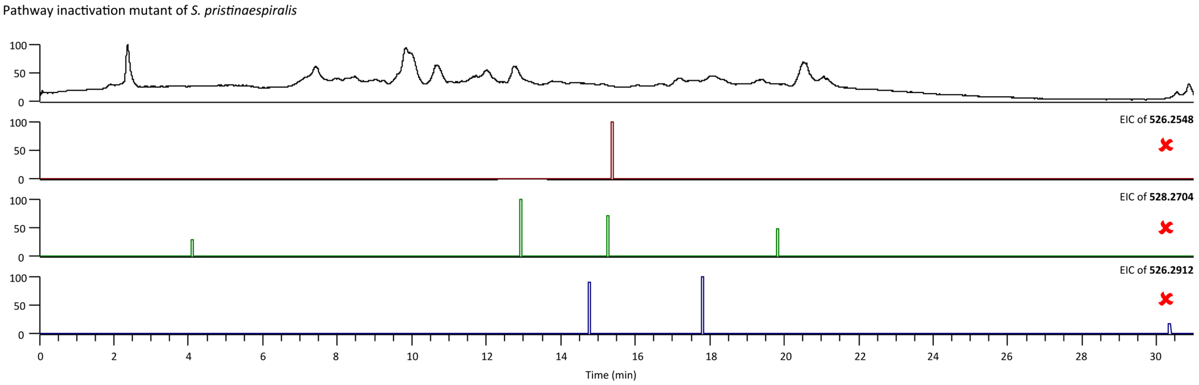

Supplementary Fig. 10 | Comparison of the metabolic spectrum of *S. pristinaespiralis* wild type with that of the pathway inactivation mutant. a. Analysis of *S. pristinaespiralis* wild type. **b.** Analysis of the pathway inactivation mutant. For each strain, the EICs of the exact masses corresponding to metabolites **1**–**3** are shown. Note: the retention times for **1**–**3** do not correspond to those reported elsewhere in the ms (i.e. **Supplementary Table 5**), as the metabolites eluted differently following replacement of the original HPLC column (see **Methods**).

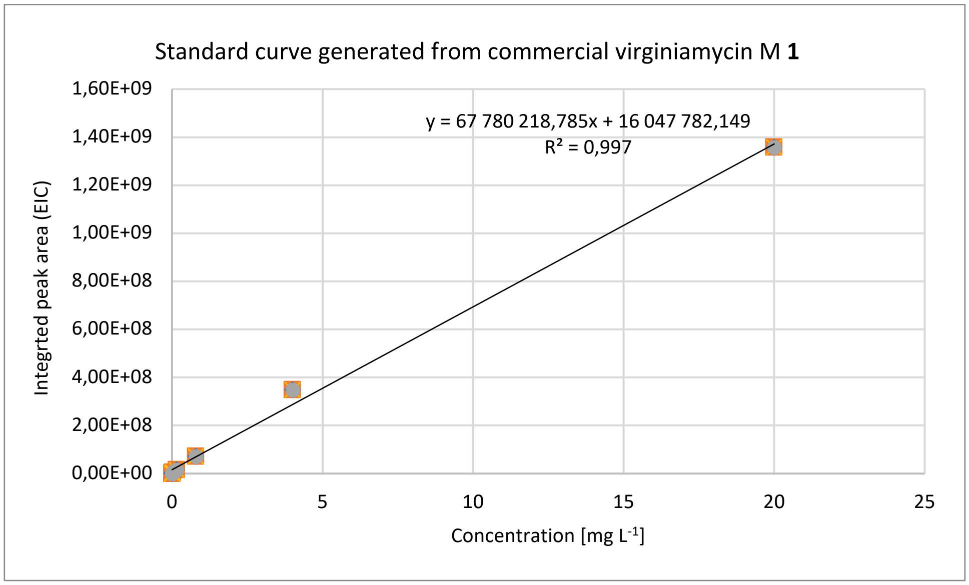

Supplementary Fig. 11 | Standard curve of commercial virginiamycin M 1 **for quantification of metabolites** 1–3**.** Accurate quantification of Vir M **1** was achieved by generation of a standard curve based on commercial Vir M, and was used to estimate the yields of the closely structurally-related metabolites **2** and **3** (**Supplementary Table 5**). Peak areas corresponding to **1** were calculated from the extracted ion chromatograms (EICs) using $[M+H]^+$ = 526.2548. A dilution series within the concentration range of 0.00000026–0.02 mg mL^{-1} was analysed. The integrated area of the 0.00000026 dilution sample was out of the linear range, and so was not included in the final data treatment. This procedure produced an essentially linear standard curve (R^2 = 0.997), as shown, for conversion of peak areas into titers (the obtained yields were then divided by 200 to correct for the concentration factor during sample preparation for HPLC-MS). Source data are provided as a Source Data file.

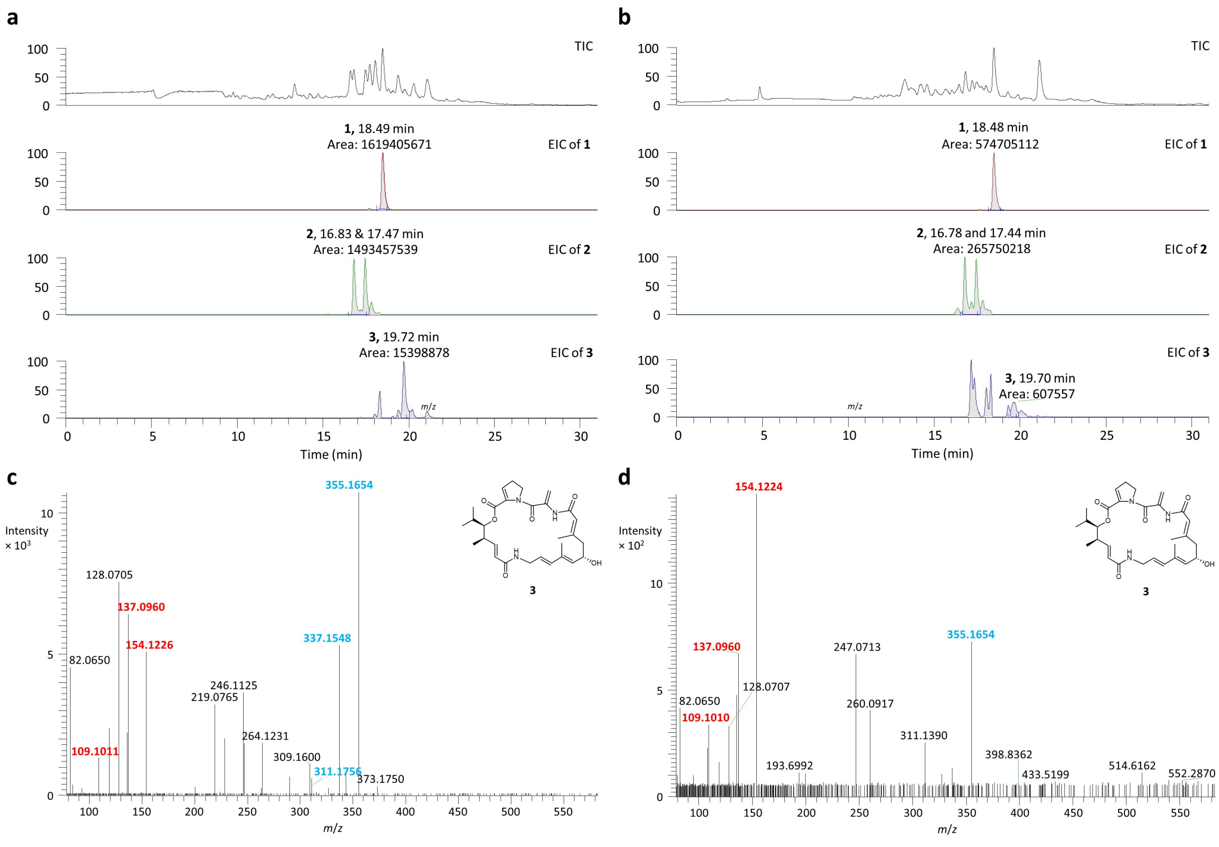

Supplementary Fig. 12 | Comparative MS/MS analysis of metabolites 1−3 **from *S. pristinaespiralis* and *S. virginiae*. a.** Analysis of *S. pristinaespiralis* extracts. Total ion chromatogram (TIC) and extracted ion chromatograms (EICs) based on the calculated accurate masses for metabolites **1**−**3**. In each case, the retention time and integrated peak areas (peaks defined by the vertical blue lines) are indicated. The retention times of **1**−**3** differ relative to those in **Fig. 5** due to a change in column (see **Methods**), as we wished to better separate metabolites **1** and **3**. We do not have a rigorous explanation for the presence of two peaks corresponding to **2**, beyond that two (stereo)isomers are present and are separated under these chromatographic conditions. **b.** Analysis of *S. virginiae* extracts. Total ion chromatogram (TIC) and extracted ion chromatograms (EICs) based on the calculated accurate masses for metabolites **1**−**3**. In each case, the retention time and integrated peak areas (peaks defined by the blue lines) are indicated. For *S. virginiae*, the peak areas have been corrected for the factor (3-fold) by which the extracts were enriched relative to those of *S. pristinaespiralis*, in order to allow for detection of **3**. **c.** MS^2 fragmentation of metabolite **3** present in extracts of *S. pristinaespiralis* (analysis shown in **a.**). Fragment colour coding is the same as that in **Fig. 6**, and the two sets of data are comparable. **d.** MS^2 fragmentation of metabolite **3** present in extracts of *S. virginiae*. Fragment colour coding is the same as that in **Fig. 6**.

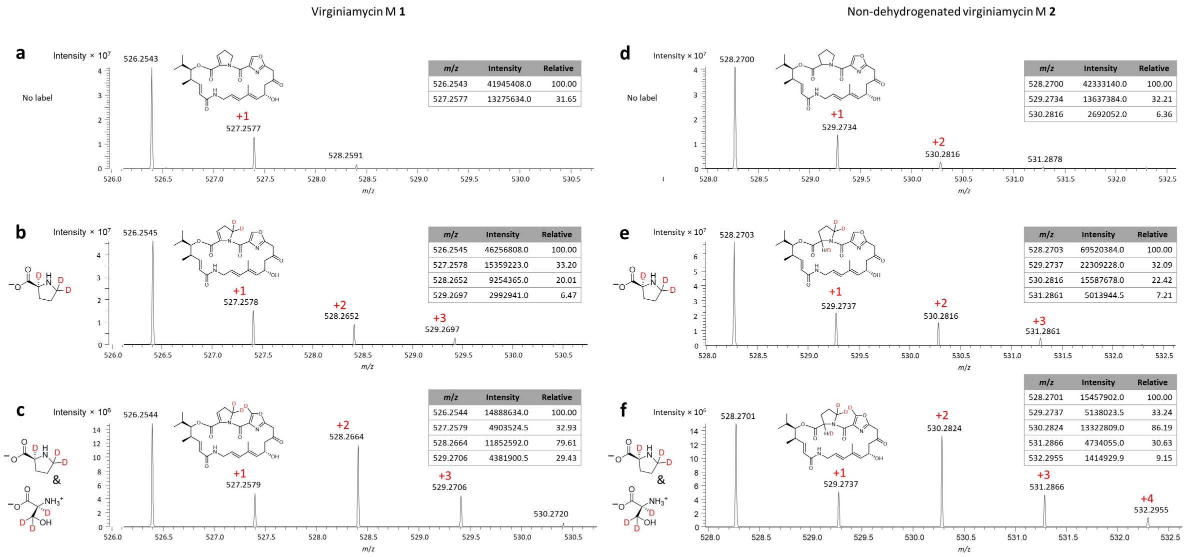

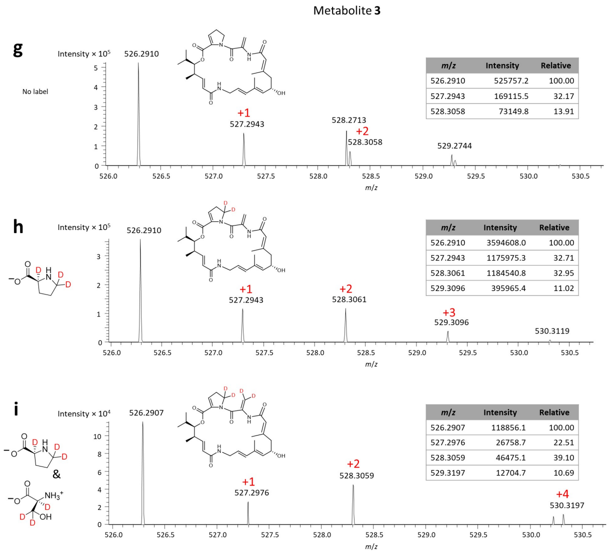

j

**1** [M+H]$^+$	526.2548	**2** [M+H]$^+$	528.2704	**3** [M+H]$^+$	526.2912
+1 (^{13}C)	527.2581	+1 (^{13}C)	529.2737	+1 (^{13}C)	527.2945
+1 (1D)	527.2611	+1 (1D)	529.2767	+1 (1D)	527.2975
+2 (^{13}C + 1D)	528.2644	+2 (^{13}C + 1D)	530.2800	+2 (^{13}C + 1D)	528.3008
+2 (2D)	528.2674	+2 (2D)	530.2830	+2 (2D)	528.3038
+3 (^{13}C + 2D)	529.2707	+3 (^{13}C + 2D)	531.2863	+3 (^{13}C + 2D)	529.3071
+3 (3D)	529.2737	+3 (3D)	531.2893	+3 (3D)	529.3101
+4 (^{13}C + 3D)	530.2770	+4 (^{13}C + 3D)	532.2926	+4 (^{13}C + 3D)	530.3134
		+4 (4D)	532.2956	+4 (4D)	530.3164

Supplementary Fig. 13 | Patterns of isotope incorporation into metabolites 1–3 **in the presence of labelled amino acids.** These experiments were conducted independently of those shown in **Fig. 7**, and thus are complementary. **a.** Mass spectrum of **1** ([M+H$^+$]) obtained in the absence of feeding (the calculated masses for **1**–**3** are presented in **Supplementary Table 5**). **b.** Mass spectrum of **1** ([M+H$^+$]) obtained in the presence of fed L-proline-2,5,5-D_3 (the deuterated positions in the Pro and in **1** are shown in red). **c.** Mass spectrum of **1** ([M+H$^+$]) obtained in the presence of fed L-proline-2,5,5-D_3 and L-serine-2,3,3-D_3. **d.** Mass spectrum of **2** ([M+H$^+$]) obtained in the absence of feeding. **e.** Mass spectrum of **2** ([M+H$^+$]) obtained in the presence of fed L-proline-2,5,5-D_3. **f.** Mass spectrum of **2** ([M+H$^+$]) obtained in the presence of fed L-proline-2,5,5-D_3 and L-serine-2,3,3-D_3. **g.** Mass spectrum of **3** ([M+H$^+$]) obtained in the absence of feeding. **h.** Mass spectrum of **3** ([M+H$^+$]) obtained in the presence of fed L-proline-2,5,5-D_3. **i.** Mass spectrum of **3** ([M+H$^+$]) obtained in the presence of fed L-proline-2,5,5-D_3 and L-serine-2,3,3-D_3. In panels **a.**–**i.**, the relative peak intensities are shown in inset. As discussed in **Fig. 7**, at the resolution at which these experiments were performed, it was not possible to distinguish the presence of ^{13}C in any given species, from the incorporation of deuterium. To reflect this ambiguity, the peaks in the spectra have been labelled +1, +2, etc. to indicate that they potentially represent a mixture of isotopically-labelled species arising from natural abundance ^{13}C and incorporation of deuterium (the respective calculated masses are shown in panel **j**.). Nonetheless, the presence of the labelled amino acids is clearly demonstrated by: i) the increase in intensity of the +2 peaks in the presence of L-proline-2,5,5-D_3 (accompanied by a +3 peak), and ii) the appearance of +4 peaks in the case of **2** and **3** when both L-serine-2,3,3-D_3 and L-proline-2,5,5-D_3 are fed. The potential +1D peak observed for metabolite **3** may arise from conversion in the cell of L-serine-2,3,3-D_3 to Gly-2-D_1 by an endogenous serine hydroxymethyl transferase (SHMT), although simultaneous incorporation of L-proline-2,5,5-D_3 and Gly-2-D_1 is not observed.

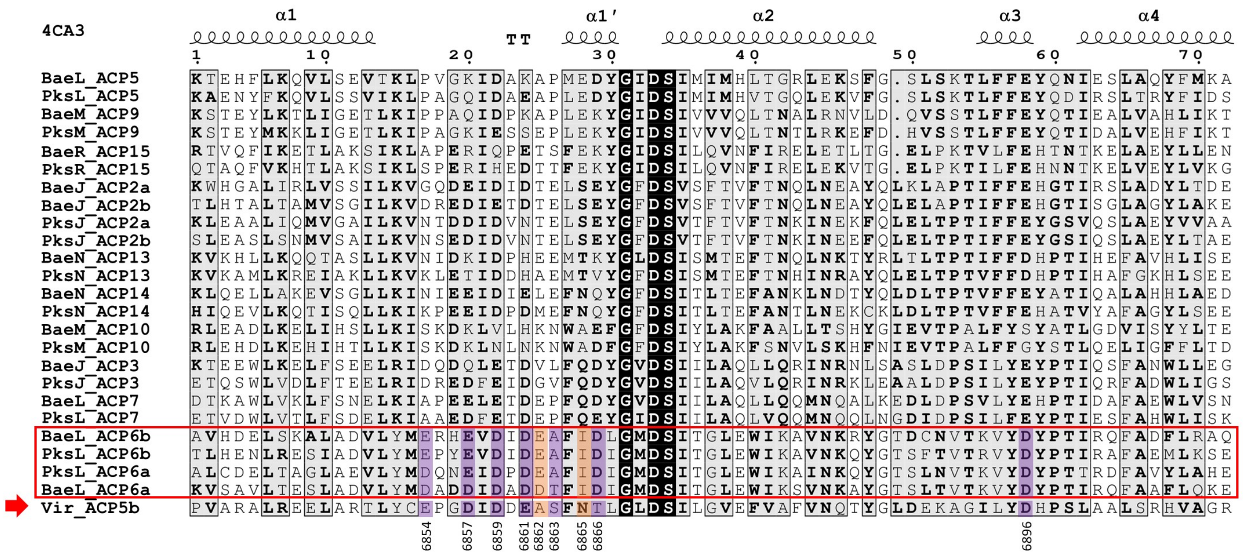

Supplementary Fig. 14 | **Analysis of Bae/Pks ACPs.** Multiple sequence alignment of all ACPs from the bacillaene (Bae)[14] and Pks[15] *trans*-AT PKS systems sourced from modules which are active for chain extension. The ACPs present in module 6 of each system targeted for β-methylation are boxed. Here, the labels 'a' and 'b' refer to the first and second domains, respectively, of tandem ACPs. The sequence of VirA ACP_{5b} is shown for comparison (red arrow) with the key interaction specificity residues highlighted in orange (loop α1-α2 amino acids) and purple (residues contributing to the domain surface potential). Sequence numbering (bottom) is based on VirA ACP_{5b}[7], and the indicated secondary structure elements are those present in the NMR structure of Vir apo-ACP_{5b} (PDB ID: 4CA3 [http://doi.org/10.2210/pdb4CA3/pdb][6]). This analysis reveals that the key specificity residues are highly similar between the two β-methylation ACPs. Abbreviations: Vir, virginiamycin; ACP, acyl carrier protein. Subunit accession numbers: BaeJ, GenBank: WP_012117595.1; BaeL, GenBank: WP_222836028.1; BaeM, GenBank: WP_012117597.1; BaeN, GenBank: WP_012117598.1; BaeR, GenBank: WP_012117599.1; PksJ, GenBank: AQR81699.1; PksL, GenBank: AQR81700.1; PksM, GenBank: AQR81701.1; PksN, GenBank: AQR81703.1; PksR, GenBank: AQR81704.1.

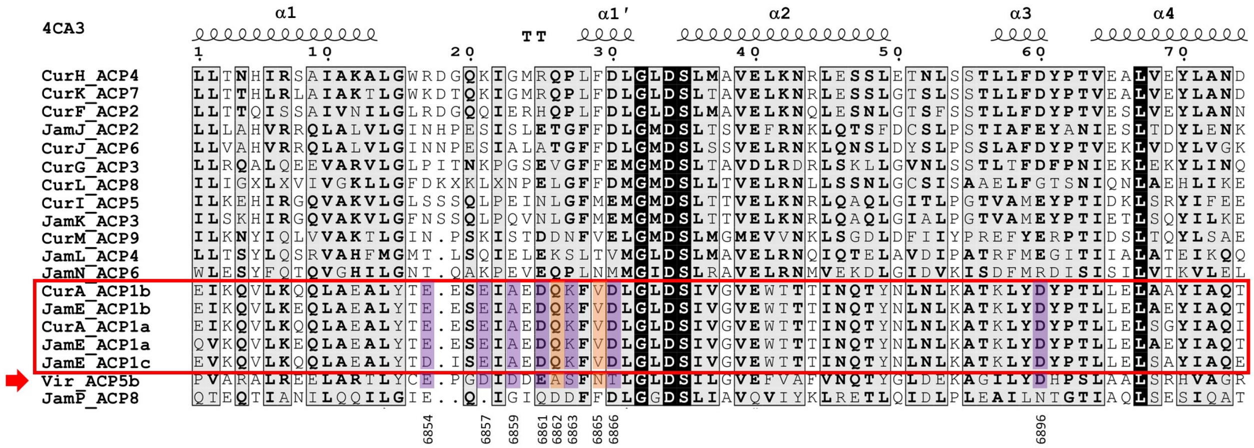

Supplementary Fig. 15 | Analysis of Cur/Jam ACPs. Multiple sequence alignment of all ACPs from the curacin (Cur)[16] and jamaicamide (Jam)[17] *cis*-AT PKSs which are present in modules which participate in chain extension. The ACPs present in module 1 of each system targeted for β-methylation are boxed. Here, the labels 'a', 'b' and 'c' refer to the first, second and third domains, of tandem ACPs. The sequence of VirA ACP_{5b} is shown for comparison (red arrow) with the key interaction specificity residues highlighted in orange (loop α1-α2 amino acids) and purple (residues contributing to the domain surface potential). Sequence numbering (bottom) is based on VirA ACP_{5b}[7]. The indicated secondary structure elements are those present in the NMR structure of Vir apo-ACP_{5b} (PDB ID: 4CA3 [http://doi.org/10.2210/pdb4CA3/pdb][6]). This analysis reveals that the key specificity residues are highly similar between the β-methylation ACPs of each system. Abbreviations: Vir, virginiamycin; ACP, acyl carrier protein. Subunit accession numbers: CurA, GenBank: AAT70096; CurF, GenBank: AAT70101.1; GurG, GenBank: AAT70102; CurH, GenBank: AAT70103; CurI, GenBank: AAT70104; CurJ, GenBank: AAT70105; CurK, GenBank: AAT70106; CurL, GenBank: AAT70107; CurM, GenBank: AAT70108; JamE, GenBank: AAS98777; JamJ, GenBank: AS98781; JamK, GenBank: AAS98782; JamL, GenBank: AAS98783; JamN, GenBank: AAS98785; JamP, GenBank: AAS98787.

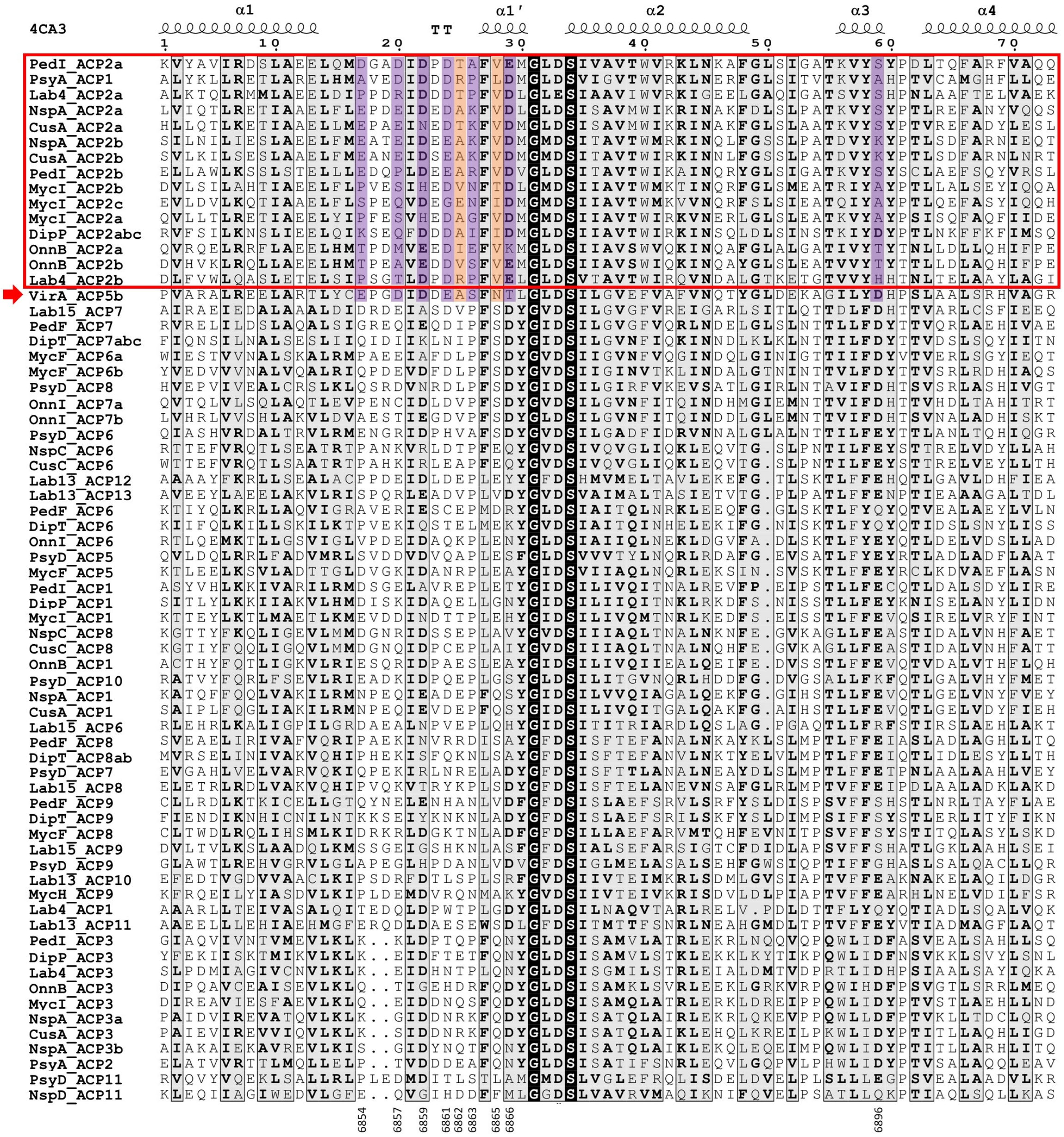

Supplementary Fig. 16 | Analysis of ACPs from the pederin family of *trans*-AT PKSs. Multiple sequence alignment of all ACPs from the pederin[18] family of *trans*-AT PKSs, which includes psymberin (Psy)[19], labrenzin (Lab)[20], nosperin (Nsp)[21], cusperin (Cus)[22], mycalamid (Myc)[23], diaphorin (Dip)[24], and onnamide (Onn)[25]. Only those ACPs from modules which are active for chain extension have been included. The ACPs present in the module of each system targeted for β-methylation are boxed. Here, the labels 'a', 'b' and 'c' refer to the first, second and third domains, of tandem ACPs, while 'ab' or 'abc' indicate that the respective ACPs have identical sequences. The sequence of VirA ACP_{5b} is shown for comparison (red arrow) with the key interaction specificity residues highlighted in orange (loop α1-α2 amino acids) and purple (residues contributing to the domain surface potential). Sequence numbering is based on VirA ACP_{5b}[7]. The indicated secondary structure elements are those present in the NMR structure of Vir apo-ACP_{5b} (PDB ID: 4CA3 [http://doi.org/10.2210/pdb4CA3/pdb][6]). This analysis reveals that the key specificity residues diverge among the β-methylation ACPs of the systems (see **Supplementary Table 6**). Abbreviations: Vir, virginiamycin; ACP, acyl carrier protein. Subunit accession numbers: PsyA, GenBank: ADA82581.1; PsyD, GenBank: ADA82585.1; Lab4, Lab13 and Lab15, GenBank: CP041191.1; NspA, GenBank: ADA69237.1; NspC, GenBank: ADA69239.2; NspD, GenBank: ADA69241.1; CusA, GenBank: AVR48533.1; CusC, GenBank: AVR48535.1; MycI, GenBank: DAC82007.1; MycF, GenBank: DAC82014.1; MycH, GenBank: DAC82016.1; DipP, GenBank: AGS06823.1; DipT, GenBank: AGS06887.1; OnnB, GenBank: AAV97870.1; OnnI, GenBank: AAV97877.1.

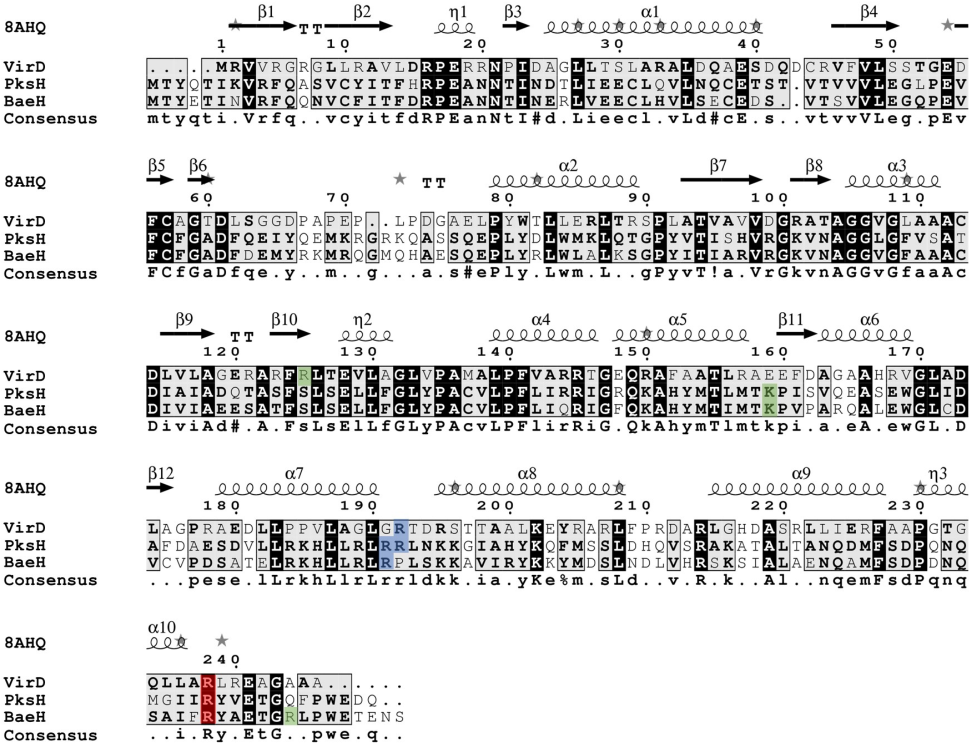

Supplementary Fig. 17 | Analysis of VirD homologues from the Bae/Pks *trans*-AT PKSs. Multiple sequence alignment of VirD with its counterparts from the bacillaene (Bae)[14] and Pks[15] systems (sequence numbering is based on VirD[7]). The indicated secondary structure elements are those present in the crystal structure of the holo-ACP_{5b}–VirD complex (PDB ID: 8AHQ [http://doi.org/10.2210/pdb8AHQ/pdb]). Residues critical for the interaction between VirD and holo-ACP_{5b} have been highlighted in colour. R125 (green) of VirD interacts with the Ppant arm. While it is not conserved in BaeH and PksH, the sequences include compensating positively-charged residues at positions 159 (both BaeH and PksH) and 244 (BaeH). Residue R192 (blue), which also contributes to Ppant positioning, contacts the highly-conserved ACP D6870 within the active site DSX motif. An Arg is present at the corresponding location in PksH, while both PksH and BaeH include Arg191, which would also be oriented correctly to interact with D6870. All three sequences incorporate R238 (red), which in the holo-ACP_{5b}–VirD complex contacts E6876 on helix $\alpha3$. Abbreviation: Vir, virginiamycin. Subunit accession numbers: PksH, GenBank: AQR85911.1; BaeH, GenBank: AQT19722.1.

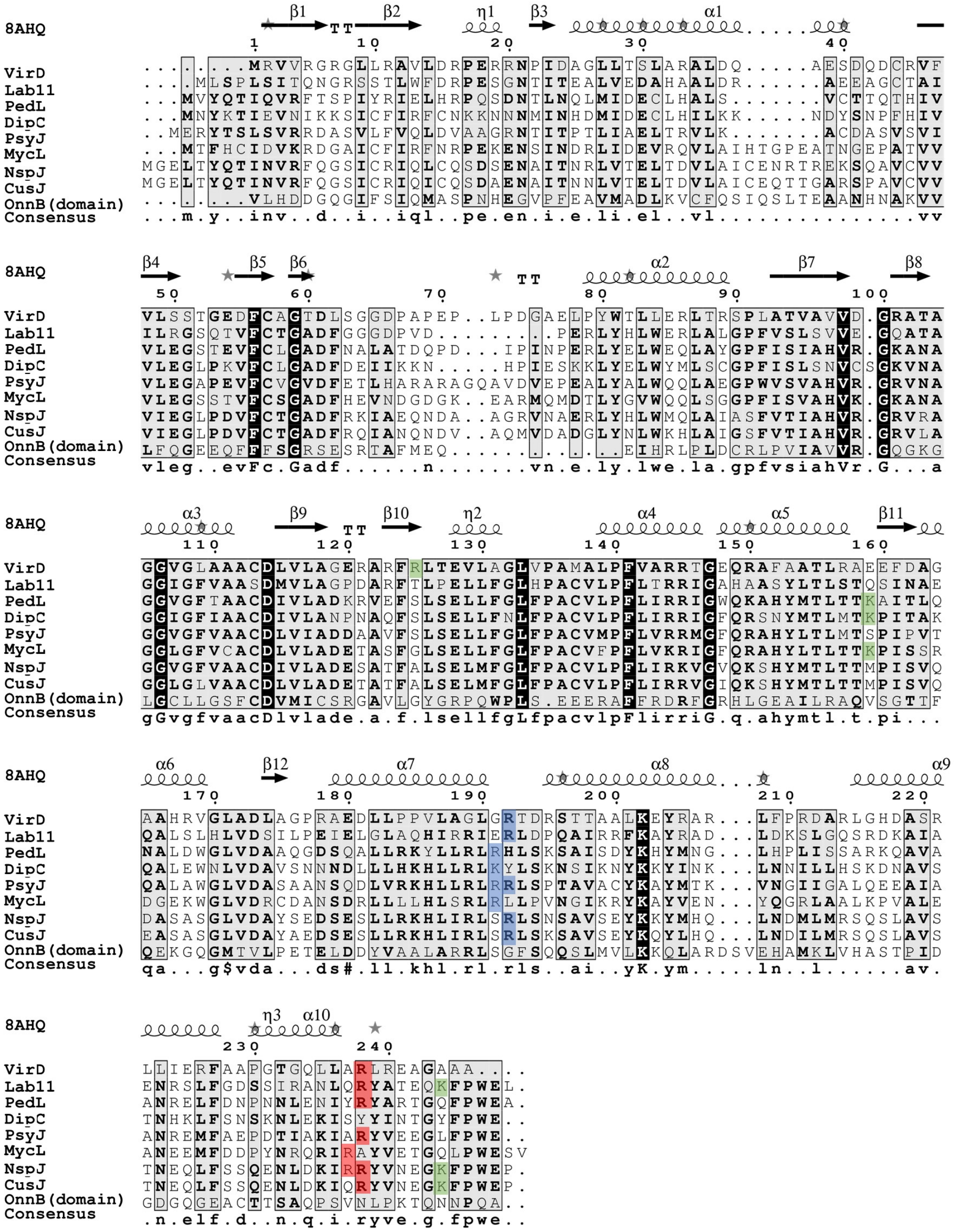

Supplementary Fig. 18 | Analysis of VirD homologues from the pederin family of *trans*-AT PKSs. Multiple sequence alignment of VirD with its counterparts from the labrenzin (Lab)[20], diaphorin (Dip)[24], psymberin (Psy)[19], mycalamid (Myc)[23], nosperin (Nsp)[21], cusperin (Cus)[22], and onnamide (Onn)[25] systems (sequence numbering is based on VirD[7]). The onnamide (Onn) homologue is present as a domain within the PKS subunit OnnB. The indicated secondary structure elements are those present in the crystal structure of the holo-ACP$_{5b}$–VirD complex (PDB ID: 8AHQ [http://doi.org/10.2210/pdb8AHQ/pdb]). Residues critical for the interaction between VirD and holo-ACP$_{5b}$ have been highlighted in colour. R125 (green) is not present in any of the pederin family sequences, but is compensated for in six cases by positively-charged lysine residues (positions 159 and 244). All sequences but OnnB contain either R192 and/or a positively-charged residue in position 191. With the exceptions of DipC and OnnB, an Arg is present at position 238 or just upstream. The absence of specificity motifs in the OnnB homologue may be explained by the fact that this domain interacts with its ACP partner(s) in *cis*. Abbreviation: Vir, virginiamycin. Subunit accession numbers: Lab11, GenBank: QDG75031.1; PedL, GenBank: AAW33971.1; DipC, GenBank: BCG49556.1; PsyJ, GenBank: ADA82590.1; MycL, GenBank: DAC82018.1; NspJ, GenBank: AVH63673.1; CusJ, GenBank: AVR48541.1; OnnB, GenBank: AAV97870.1.

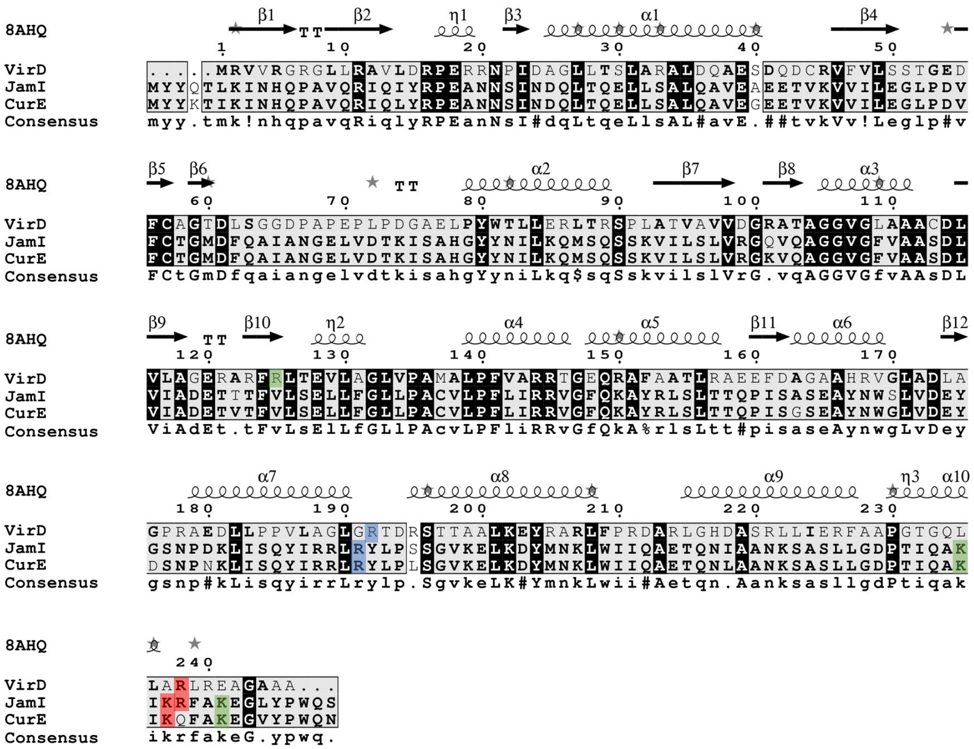

Supplementary Fig. 19 | Analysis of VirD homologues from the Cur/Jam *cis*-AT PKSs. Multiple sequence alignment of VirD with its counterparts from the curacin (Cur)[16] and jamaicamide (Jam)[17] systems (sequence numbering is based on VirD[7]). The indicated secondary structure elements are those present in the crystal structure of the holo-ACP_{5b}–VirD complex (PDB ID: 8AHQ [http://doi.org/10.2210/pdb8AHQ/pdb]). Residues critical for the interaction between VirD and holo-ACP_{5b} have been highlighted in colour. Neither homologue includes R192 (blue), but Arg residues are present at 191 in both sequences. Likewise, K237 in each sequence may replace and/or complement R238 (red). While no evident compensation is provided for the lack of R125 (green), the presence of multiple Lys residues in the flexible C-terminus of the protein, suggests that they may furnish the missing positive charges if helix α10 is positioned differently. Abbreviation: Vir, virginiamycin. Subunit accession numbers: JamI, GenBank: AAS98780.1; CurE, GenBank: AAT70100.1.

Supplementary Table 1 │ List of strains used in this work.

Strains/mutants	Description and use (resistance)	Reference
S. pristinaespiralis		
ATCC	*S. pristinaespiralis* ATCC 25486 wild type strain	DMSZ
Pathway mutant	Inactivation mutant at the Sna module 7/module 8 interface) ($Apra^R$)	This work
S. virginiae		
MAFF No. 116014	*S. virginiae* MAFF No. 116014 wild type strain	Genebank Project, National Institute of Agrobiological Sciences, Japan
E. coli		
DH5α	General cloning strain	[26]
ET12567/pUZ8002	Non-methylating strain containing a mobilisation plasmid for conjugation with *Streptomyces* (Kan^R, Cm^R)	[27]
BL21	BL21 (DE3), a widely used T7 expression strain	Novagen
BL21/pLM-302_VirC	Strain for VirC overexpression (Kan^R)	This work
BL21/pLM-302_ VirC C114A/Q334A/R335A/R338A	Strain for VirC C114A/Q334A/R335A/R338A overexpression (Kan^R)	This work
BL21/pBG-102_VirD	Strain for VirD overexpression (Kan^R)	This work
BL21/pBG-102_VirD E128Q	Strain for VirD E128Q overexpression (Kan^R)	This work
BL21/pBG-102_VirE	Strain for VirE overexpression (Kan^R)	This work
BL21/pBG-102_ACP_{5a}	Strain for ACP_{5a} overexpression (Kan^R)	This work
BL21/pBG-102_ACP_{5b}	Strain for ACP_{5b} overexpression (Kan^R)	This work
BL21/pBG-102_ACP_{5a}-ACP_{5b}	Strain for ACP_{5a}-ACP_{5b} overexpression (Kan^R)	This work
BL21/pBG-102_ACP_{5a} E6761A	Strain for ACP_{5a} E6761A overexpression (Kan^R)	This work
BL21/pBG-102_ACP_{5a} L6764N	Strain for ACP_{5a} L6764N overexpression (Kan^R)	This work
BL21/pBG-102_ACP_{5a} E6761A/L6764N	Strain for ACP_{5a} E6761A/L6764N overexpression (Kan^R)	This work
BL21/pBG-102_ACP_6	Strain for ACP_6 overexpression (Kan^R)	This work
BL21/pBG-102_ACP_7	Strain for ACP_7 overexpression (Kan^R)	This work
BL21/pBG-102_Sna ACP_7	Strain for Sna ACP_7 overexpression (Kan^R)	This work

Supplementary Data Table 2 | Summary of SAXS data.

	holo-ACP$_{5b}$–VirC	VirD	holo-ACP$_{5b}$–VirD	VirE	holo-ACP$_{5b}$–VirE
**Structural parameters from Guinier fits, P(r) functions, MW estimates**					
**Guinier analysis**					
$I(0)$ (cm^{-1})	0.10 ± 0.00015	0.11 ± 0.00017	0.06 ± 0.00017	0.28 ± 0.00047	0.16 ± 0.00018
R_g (Å)	29.28 ± 0.06	28.68 ± 0.07	29.69 ± 0.12	32.21 ± 0.08	31.80 ± 0.06
q_{min} (Å^{-1})	0.0005	0.0005	0.0005	0.0005	0.0002
qR_g max (Å^{-1})	1.3	1.3	1.3	1.3	1.3
**P(r) analysis**					
$I(0)$ cm^{-1}	0.1016 ± 0.1176E^{-03}	0.1091 ± 0.1421E^{-03}	0.0671 ± 0.1826E^{-03}	0.2823 ± 0.4133E^{-03}	0.1555 ± 0.1828E^{-03}
R_g (Å)	29.20 ± 0.04	28.54 ± 0.03	29.88 ± 0.13	33.01 ± 0.07	31.79 ± 0.16
D_{max} (Å)	101.69	88.95	110.50	121.90	110.53
q range (Å^{-1})	0.02 - 0.2730	0.01 - 0.27845	0.01 - 0.2694	0.02 - 0.2480	0.01 - 0.2516
χ^2 (total estimate from GNOM)	0.8989	0.9455	0.6817	0.7875	0.8859
Porod Volume (Å^{-3}) (ratio V_p/calculated M)	141215	130507	131379	139351	140370
**Estimation of the Molecular Weight (MW)**					
MW from chemical composition (kDa)	110.66	78.92	107.57	81.37	110.02
MW (Bayesian inference) (kDa)	85.650	74.325	80.750	94.225	94.225
Credibility interval (kDa)	79.60 – 89.70	71.45 –79.60	75.30 – 84.35	86.95 – 95.80	89.70 – 99.20
MW probability (%), Credibility interval (%)	40.89, 97.53	49.95, 92.95	52.52, 94.04	51.23, 92.43	61.73, 90.14
Oligomeric state	tetramer	trimer	hexamer	trimer	hexamer
**Report modelling results**					
	**Crystal structures**			**Modelled structures**	
**CRYSOL with default parameters (No constant subtraction)**			**OLIGOMER**	**CORAL**	**SASREF**
Structure	5KP6	8AHZ	VirD/holo-ACP$_{5b}$–VirD	VirE with disordered C-ter	ACP$_{5b}$–VirE
χ^2	1.52	2.10	1.72	1.65	1.89
Predicted R_g (Å)	29.09	25.67	29.30	33.03	31.80
Predicted envelope diameter (Å)	107.10	84.32	n.d	n.d	n.d
Vol (Å), Ra (Å), Dro (e Å^{-3})	143593, 1.74, 0.05	101814, 1.80, 0.00	n.d	n.d	n.d

n.d. = not determined

Supplementary Data Table 3 | Data collection, phasing and refinement statistics for SAD (SeMet) structures.

	Se-VirD high resolution	Se-VirD	holo-ACP_{5b}–VirD
**Data collection**			
Space group	P 4_12_12	P 4_12_12	H3
Cell dimensions			
a, *b*, *c* (Å)	84.31, 84.31, 230.19	84.31, 84.31, 230.19	144.88, 144.88, 88.78
α, β, γ (°)	90.00, 90.00, 90.00	90.00, 90.00, 90.00	90.00, 90.00, 120.00
		Peak	
Wavelength	0.953709	0.979260	0.95370
Resolution (Å)	59.62-1.76 (1.81-1.76)	47.41-2.02 (2.07-2.02)	36.23-2.10 (2.16-2.10)
R_{merge} (%)	7.1 (75.3)	13.3 (87.9)	12.5 (146.7)
R_{pim} (%)	2.9 (31.5)	2.6 (18.2)	3.7 (45.2)
$I / \sigma I$	19.5 (2.4)	20.3 (3.5)	15.5 (3.0)
Number of total observations	1027674	1425966	479439
Number of unique reflections	81872	54825	40571
Completeness (%)	99.0 (85.9)	99.6 (94.9)	100 (100)
Redundancy	12.6 (11.6)	26.0 (22.7)	11.8 (11.1)
Wilson *B*-factors (Å^2)	26.5	n.d.	40.8
**Refinement**			
Resolution (Å)	57.78-1.70		41.85-2.10
No. reflections	6616		40571
R, R_{free}	0.159, 0.180		0.183, 0.238
No. atoms	5851		5127
Protein	5261		4736
Ligand/ion	152		69
Water	438		322
Average B-factors, all atoms (Å^2)	29.0		39.0
R.m.s deviations			
Bond lengths (Å)	0.0086		0.0123
Bond angles (°)	1.5167		1.7890

n.d. = not determined

Supplementary Table 4 | NMR and refinement statistics for protein structures.

	ACP_{5a}	ACP_6	ACP_7
**NMR distance and dihedral constraints**			
Distance constraints			
Total NOE	1822	1208	1763
Intra-residue	379	376	409
Inter-residue	1443	832	1354
Sequential ($\|i - j\| = 1$)	444	349	476
Medium-range ($\|i - j\| < 4$)	521	267	488
Long-range ($\|i - j\| > 5$)	478	216	390
Intermolecular	0	0	0
Hydrogen bonds	0	0	0
Total dihedral angle restraints			
ϕ	55	46	47
ψ	55	46	47
**Structure statistics**			
Violations (mean and s.d.)			
Distance constraints (Å)	0.064 ± 0.012	0.088 ± 0.033	0.088 ± 0.023
Dihedral angle constraints (º)	0 ± 0	0 ± 0	0 ± 0
Max. dihedral angle violation (º)	0	0	0
Max. distance constraint violation (Å)	0.09	0.14	0.13
Deviations from idealised geometry			
Bond lengths (Å)	0.010 ± 5.53E-5	0.011 ± 7.68E-5	0.011 ± 9.78E-05
Bond angles (º)	2.046 ± 0.014	2.112 ± 0.019	2.169 ± 0.016
Impropers (º)	N/A	N/A	N/A
Average pairwise r.m.s. deviation** (Å)	Residues 6734−6809	Residues 1082−1158	Residues 1877−1944
Heavy	0.43 ± 0.08	0.87 ± 0.13	0.53 ± 0.09
Backbone	1.23 ± 0.15	1.71 ± 0.14	1.11 ± 0.12

**Pairwise r.m.s. deviation was calculated among 20 refined structures.

Supplementary Table 5 | Spectroscopic and yield data for metabolites 1−3**.**

Compound	Chemical formula	Retention time (min)	Calculated m/z ratio [M+H]+	Observed m/z ratio [M+H]+	Major MS^2 fragments[a]	Integrated peak areas[b]	Calculated titer (mg L^{-1})[c]
Virginiamycin M (Vir M) **1**	$C_{28}H_{35}N_3O_7$	12.50−12.66	526.2548	526.2549 (+0.38 ppm)	508.2441, 395.1968, 355.1289, 337.1184, 311.1392, 260.0918, 242.0812, 231.0401, 205.0608, 154.1225, 150.0912, 137.0960, 133.0647, 114.0549, 109.1011	1792296235<br>1670983468<br>1275350367	0.12 ± 0.02
Non-dehydrogenated Vir M **2**	$C_{28}H_{37}N_3O_7$	11.69−11.99	528.2704	528.2701 (−0.57 ppm)	510.2598, 357.1452, 339.1352, 321.1234, 311.1391, 295.1442, 244.0968, 154.1225, 137.0962, 135.0804, 109.1011	1097693947<br>1017328497<br>1463514063	0.09 ± 0.01
Doubly β-methylated derivative **3**	$C_{29}H_{39}N_3O_6$	13.35−13.71	526.2912	526.2909 (−0.57 ppm)	355.1653, 337.1549, 311.1756, 264.1231, 246.1125, 228.1019, 219.0765, 154.1226, 137.0960, 128.0705, 109.1011, 82.0650	27013749<br>19879608<br>26359953	0.0006 ± 0.0003

[a]Orange: fragments differing by 2 Da between **1** and **2**, and thus likely containing the Pro/2-pyrroline residue; green: fragments common to **1** and **2** and not observed with **3**; red: fragments common to **1**, **2** and **3**; blue: fragments of **3** which exhibit the same increase in mass relative to the corresponding fragments of **1** as that seen between the parental molecules **3** and **1**.

[b]The data were obtained from three biological replicates. The integrated peak areas were converted to titres using the calculation described in **Supplementary Fig. 11**.

[c]While yields of all three metabolites were substantially improved by addition of XAD-16 resin (by ≥ 25−50-fold, bringing them closer to those previously reported for *S. pristinaespiralis* ATCC25486 (13 mg L^{-1})[28]), the clearest labelling results were obtained in its absence, making the presented titres most relevant.

Supplementary Table 6│ Analysis of α1-α2 loop residues in pederin family ACPs targeted for β-modification

System	Critical residues			Prediction: in-parallel/in-series	ACP_{2b} pair of residues present in any other ACPs?
	ACP_{2a}	ACP_{2b}	ACP_{2c}		
Vir	EL	AN		In-series[a]	N
Lab	TV	RV		In-series	N
Onn	IV	VV		In-parallel[b]	N
Dip	AI	AI	AI	In-parallel	N
Myc	AV	VT	EI	In-series	N
Psy	RL			N.A. (one ACP)	N
Ped	TV	AV		In-series	Y (PedF ACP_9)
Cus	TV	AV		In-series	N
Nsp	AV	AV		In-parallel	N

[a]Demonstrated directly in this study.

[b]The ability of the two ACPs to act in-parallel may be limited by their covalent attachment to the ECH_1 and ECH_2 homologues within the context of subunit OnnB[25].

Supplementary References

1. Svergun, D., Barberato, C. & Koch, M. H. J. CRYSOL – a program to evaluate X-ray solution scattering of biological nacromolecules from atomic coordinates. *J. Appl. Crystallogr.* **28**, 768–773 (1995).
2. Konarev, P. V., Volkov, V. V., Sokolova, A. V., Koch, M. H. J. & Svergun, D. I. PRIMUS: a Windows PC-based system for small-angle scattering data analysis. *J. Appl. Crystallogr.* **36**, 1277–1282 (2003).
3. Mirdita, M. *et al.* ColabFold: making protein folding accessible to all. *Nat. Methods* **19**, 679–682 (2022).
4. Petoukhov, M. V. *et al.* New developments in the ATSAS program package for small-angle scattering data analysis. *J. Appl. Crystallogr.* **45**, 342–350 (2012).
5. Petoukhov, M. V. & Svergun, D. I. Global rigid body modeling of macromolecular complexes against small-angle scattering data. *Biophys. J.* **89**, 1237–1250 (2005).
6. Davison, J. *et al.* Insights into the function of *trans*-acyl transferase polyketide synthases from the SAXS structure of a complete module. *Chem. Sci.* **5**, 3081–3095 (2014).
7. Haines, A. S. *et al.* A conserved motif flags acyl carrier proteins for β-branching in polyketide synthesis. *Nat. Chem. Biol.* **9**, 685–692 (2013).
8. Pan, G. *et al.* Discovery of the leinamycin family of natural products by mining actinobacterial genomes. *Proc. Natl. Acad. Sci. U. S. A.* **114**, E11131–E11140 (2017).
9. Erol, O. *et al.* Biosynthesis of the myxobacterial antibiotic corallopyronin A. *ChemBioChem* **11**, 1253–1265 (2010).
10. Helfrich, E. J. N. *et al.* Automated structure prediction of *trans*-acyltransferase polyketide synthase products. *Nat. Chem. Biol.* **15**, 813–821 (2019).
11. Sucipto, H., Wenzel, S. C. & Müller, R. Exploring chemical diversity of α-pyrone antibiotics: molecular basis of myxopyronin biosynthesis. *ChemBioChem* **14**, 1581–1589 (2013).
12. Tang, G.-L., Cheng, Y.-Q. & Shen, B. Leinamycin biosynthesis revealing unprecedented architectural complexity for a hybrid polyketide synthase and nonribosomal peptide synthetase. *Chem. Biol.* **11**, 33–45 (2004).
13. Cobb, R. E., Wang, Y. & Zhao, H. High-efficiency multiplex genome editing of Streptomyces species using an engineered CRISPR/Cas system. *ACS Synth. Biol.* **4**, 723–728 (2015).
14. Moldenhauer, J., Chen, X.-H., Borriss, R. & Piel, J. Biosynthesis of the antibiotic bacillaene, the product of a giant polyketide synthase complex of the *trans*-AT family. *Angew. Chem. Int. Ed Engl.* **46**, 8195–8197 (2007).
15. Butcher, R. A. *et al.* The identification of bacillaene, the product of the PksX megacomplex in *Bacillus subtilis*. *Proc. Natl. Acad. Sci. U. S. A.* **104**, 1506–1509 (2007).
16. Chang, Z. *et al.* Biosynthetic pathway and gene cluster analysis of curacin A, an antitubulin natural product from the tropical marine cyanobacterium *Lyngbya majuscula*. *J. Nat. Prod.* **67**, 1356–1367 (2004).
17. Edwards, D. J. *et al.* Structure and biosynthesis of the jamaicamides, new mixed polyketide-peptide neurotoxins from the marine cyanobacterium *Lyngbya majuscula*. *Chem. Biol.* **11**, 817–833 (2004).
18. Piel, J. A polyketide synthase-peptide synthetase gene cluster from an uncultured bacterial symbiont of *Paederus* beetles. *Proc. Natl. Acad. Sci. U. S. A.* **99**, 14002–14007 (2002).
19. Fisch, K. M. *et al.* Polyketide assembly lines of uncultivated sponge symbionts from structure-based gene targeting. *Nat. Chem. Biol.* **5**, 494–501 (2009).
20. Kačar, D. *et al.* Genome of *Labrenzia* sp. PHM005 reveals a complete and active *trans*-AT PKS gene cluster for the biosynthesis of labrenzin. *Front. Microbiol.* **10**, 2561 (2019).
21. Kampa, A. *et al.* Metagenomic natural product discovery in lichen provides evidence for a family of biosynthetic pathways in diverse symbioses. *Proc. Natl. Acad. Sci. U. S. A.* **110**, E3129–3137 (2013).
22. Kust, A. *et al.* Discovery of a pederin family compound in a nonsymbiotic bloom-forming cyanobacterium. *ACS Chem. Biol.* **13**, 1123–1129 (2018).
23. Storey, M. A. *et al.* Metagenomic exploration of the marine sponge *Mycale hentscheli* uncovers multiple polyketide-producing bacterial symbionts. *mBio* **11**, e02997-19 (2020).
24. Nakabachi, A. *et al.* Defensive bacteriome symbiont with a drastically reduced genome. *Curr. Biol.* **23**, 1478–1484 (2013).
25. Piel, J. *et al.* Antitumor polyketide biosynthesis by an uncultivated bacterial symbiont of the marine sponge *Theonella swinhoei*. *Proc. Natl. Acad. Sci. U. S. A.* **101**, 16222–16227 (2004).
26. Hanahan, D. Studies on transformation of *Escherichia coli* with plasmids. *J. Mol. Biol.* **166**, 557–580 (1983).
27. MacNeil, D. J. *et al.* Analysis of *Streptomyces avermitilis* genes required for avermectin biosynthesis utilizing a novel integration vector. *Gene* **111**, 61–68 (1992).
28. Jin, Q., Jin, Z., Zhang, L., Yao, S. & Li, F. Probing the molecular mechanisms for pristinamycin yield enhancement in *Streptomyces pristinaespiralis*. *Curr. Microbiol.* **65**, 792–798 (2012).
