# Supplementary material for: Decrypting the programming of β-methylation in virginiamycin M biosynthesis
Source: Nat Commun. 2023 Mar 10;14:1327. doi: 10.1038/s41467-023-36974-3 (PMC10006238; doi:10.1038/s41467-023-36974-3)
Supplement: Supplementary file 3 — Description of Additional Supplementary Files [file 41467_2023_36974_MOESM3_ESM.pdf]

Description of Additional Supplementary Files

Supplementary Data 1: oligonucleotide sequences
