# Supplementary material for: Decrypting the programming of β-methylation in virginiamycin M biosynthesis
Source: Nat Commun. 2023 Mar 10;14:1327. doi: 10.1038/s41467-023-36974-3 (PMC10006238; doi:10.1038/s41467-023-36974-3)
Supplement: Supplementary file 5 — Reporting Summary [file 41467_2023_36974_MOESM5_ESM.pdf]

nature portfolio

Corresponding author(s): Chagot, Benjamin; Weissman, Kira J; Gruez, Arnaud

Last updated by author(s): Jan 26, 2023

Reporting Summary

Nature Portfolio wishes to improve the reproducibility of the work that we publish. This form provides structure for consistency and transparency in reporting. For further information on Nature Portfolio policies, see our Editorial Policies and the Editorial Policy Checklist.

Statistics

For all statistical analyses, confirm that the following items are present in the figure legend, table legend, main text, or Methods section.

n/a	Confirmed	
☐	☒	The exact sample size (*n*) for each experimental group/condition, given as a discrete number and unit of measurement
☐	☒	A statement on whether measurements were taken from distinct samples or whether the same sample was measured repeatedly
☒	☐	The statistical test(s) used AND whether they are one- or two-sided<br>*Only common tests should be described solely by name; describe more complex techniques in the Methods section.*
☒	☐	A description of all covariates tested
☒	☐	A description of any assumptions or corrections, such as tests of normality and adjustment for multiple comparisons
☒	☐	A full description of the statistical parameters including central tendency (e.g. means) or other basic estimates (e.g. regression coefficient) AND variation (e.g. standard deviation) or associated estimates of uncertainty (e.g. confidence intervals)
☒	☐	For null hypothesis testing, the test statistic (e.g. *F*, *t*, *r*) with confidence intervals, effect sizes, degrees of freedom and *P* value noted<br>*Give P values as exact values whenever suitable.*
☒	☐	For Bayesian analysis, information on the choice of priors and Markov chain Monte Carlo settings
☒	☐	For hierarchical and complex designs, identification of the appropriate level for tests and full reporting of outcomes
☒	☐	Estimates of effect sizes (e.g. Cohen's *d*, Pearson's *r*), indicating how they were calculated

Our web collection on statistics for biologists contains articles on many of the points above.

Software and code

Policy information about availability of computer code

Data collection	Fluorescence Xenius Spectrophotometer (SAFAS) for Tryptophan fluorescence quenching measurements<br>Chirascan CD (Applied Photophysics) for CD measurements<br>Synchrotron SOLEIL SWING and PROXIMA2 beamlines for SAXS and X-ray data collection<br>Thermo Scientific Orbitrap ID-X Tribrid for MS detection<br>DRX600 spectrometer equipped with a cryogenic probe (Bruker) for protein NMR data acquisition
Data analysis	ExPASy ProtParam tool for calculation of protein extinction coefficients<br>SciDAVis v2.3.0. for data fluorescence analysis<br>XDS v. June 1, 2017, for indexing and integrating X-ray diffraction data<br>CCP4 suite v. 7.1 (REFMAC5, MOLREP, ARP/wARP), COOT and Phenix.autosol for structure determination and refinement<br>MolProbity v. 4.2 for analysis and validation of sterics and geometry of proteins crystal structures<br>FOXTROT v. 3.4.1 for reduction of SAXS data to absolute units, frame averaging, and solvent subtraction<br>ATSAS suite v. 3.0 (CRYSOL, SARESF, CORAL, OLIGOMER), GNOM, PRIMUS for SAXS data analysis<br>ColabFold v. 1.4 for accelerated prediction of the structures of proteins and complexes<br>PyMOL v. 2.5.0 for visualizing 3D molecular structures and creation of high quality figures<br>Thermo Xcalibur v. 2.1 and v. 4.3 software for MS analysis<br>Topspin 3.2, NMRFAM-SPARKY and CYANA 3.98 software (TALOS-N, AMBER 14, PROCHECK-NMR) for NMR data processing and structure calculations<br>Combined use of Clustal W (https://npsa-prabi.ibcp.fr/cgi-bin/npsa_automat.pl?page=/NPSA/npsa_clustalw.html) available via the NPS@ server and ESPript (https://espript.ibcp.fr/ESPript/cgi-bin/ESPript.cgi) for amino acid sequence alignment and display

Clustal W for DNA sequence analysis
CRISPy-web software v. 2 for design of sgRNA used in CRISPR-Cas9-based genome engineering

For manuscripts utilizing custom algorithms or software that are central to the research but not yet described in published literature, software must be made available to editors and reviewers. We strongly encourage code deposition in a community repository (e.g. GitHub). See the Nature Portfolio guidelines for submitting code & software for further information.

Data

Policy information about availability of data

All manuscripts must include a data availability statement. This statement should provide the following information, where applicable:

- Accession codes, unique identifiers, or web links for publicly available datasets
- A description of any restrictions on data availability
- For clinical datasets or third party data, please ensure that the statement adheres to our policy

Crystal structures of VirD and the holo-ACP5b–VirD complex have been deposited in the Protein Data Bank with their respective diffraction data under accession codes 8AHZ [http://doi.org/10.2210/pdb8AHZ/pdb] and 8AHQ [http://doi.org/10.2210/pdb8AHQ/pdb], respectively. Coordinates and chemical shifts for the NMR structures of holo-ACP5a, holo-ACP6 and holo-ACP7 have been deposited in the Protein Data Bank with accession codes 8A7Z [http://doi.org/10.2210/pdb8A7Z/pdb], 8AIG [http://doi.org/10.2210/pdb8AIG/pdb], and 8ALL [http://doi.org/10.2210/pdb8ALL/pdb], and in the Biological Magnetic Resonance Bank with accession codes 34739 [https://bmrb.io/data_library/summary/?bmrbId=34739], 34743 [https://bmrb.io/data_library/summary/?bmrbId=34743] and 34744 [https://bmrb.io/data_library/summary/?bmrbId=34744], respectively. Raw SAXS and HPLC-MS data have been deposited in the data repository DOREL (DOnnées de la REcherche Lorraines) [https://doi-org.insis.bib.cnrs.fr/10.12763/PEYXHP] with accession code https://doi.org/10.12763/GYAWHI. The remaining data supporting this study are included in the Supplementary Information. Source data are provided with this paper, and all biological materials are available from the authors upon request.

Human research participants

Policy information about studies involving human research participants and Sex and Gender in Research.

Reporting on sex and gender	We have not carried out sex- and gender-based analysis, because our study does not involve humans, vertebrate animals or cell lines.
Population characteristics	*Describe the covariate-relevant population characteristics of the human research participants (e.g. age, genotypic information, past and current diagnosis and treatment categories). If you filled out the behavioural & social sciences study design questions and have nothing to add here, write "See above."*
Recruitment	*Describe how participants were recruited. Outline any potential self-selection bias or other biases that may be present and how these are likely to impact results.*
Ethics oversight	*Identify the organization(s) that approved the study protocol.*

Note that full information on the approval of the study protocol must also be provided in the manuscript.

Field-specific reporting

Please select the one below that is the best fit for your research. If you are not sure, read the appropriate sections before making your selection.

☒ Life sciences ☐ Behavioural & social sciences ☐ Ecological, evolutionary & environmental sciences

For a reference copy of the document with all sections, see nature.com/documents/nr-reporting-summary-flat.pdf

Life sciences study design

All studies must disclose on these points even when the disclosure is negative.

Sample size	We investigated the two ACP domains (ACP5a and ACP5b) in the only virginiamycin (Vir) PKS module thought to be targeted for beta-methylation (module 5), and selected two other ACP domains from the same system (ACP6 and ACP7) as 'controls' (the finding that ACP7 is also recognized by the beta-methylation cassette was unexpected).<br>We mutated two ACP residues to test the validity of our interaction model, as they played distinct roles in recognition (contribution to the domain surface potential and orientation of the Ppant prosthetic group within the VirD complex).<br>We investigated the virgniamycin metabolic profile in the only two Streptomyces species (S. virginiae and S. pristinaespiralis) reported to produce virginiamycin M.<br>Selection of sequences for comparative analysis of ACP/VirD recognition elements was based on two considerations: i) the availability of complementary biochemical data (the case for the bacillaene/Pks and curacin/jamaicamide systems); and ii) the large size of the pederin PKS family, which allowed for direct comparison among multiple members.
Data exclusions	No data were excluded.

Replication	Each of the fluorescence measurements was carried out in duplicate. The individual measurements are reported in Supplementary Figs. 3-5, as well as the mean values. Three independent fermentations of Streptomyces pristinaespiralis were analyzed (see Supplementary Table 5) to confirm the presence of specific metabolites and estimate their yields. As this analysis showed some variation, the average yields were calculated, which allowed for assessment of production variability. Production of metabolite 3 from Streptomyces virginiae was near the limit of detection, and so this strain was analyzed only once. Nonetheless, convincing MS and MS2 data were obtained demonstrating the identity of 3. The standard curves for quantifying the metabolites were based on one or two HPLC-MS measurements at each concentration, as we observed excellent agreement between the sample concentrations and the peak areas. The feeding experiments with isotopically-labelled amino acids were carried out independently twice with L-proline-2,5,5-D3 or L-serine-2,3,3-D3, and combined L-proline-2,5,5-D3 and L-serine-2,3,3-D3, and representative data are presented.
Randomization	Randomization was not relevant to our study, as we did not compare groups.
Blinding	No groups were compared in these study, and therefore blinding was unnecessary. All of the analytical techniques used (fluorescence quenching, circular dichroisme and MS/MS) are unbiased, and so there was no reason to suspect bias in the analysis.

Reporting for specific materials, systems and methods

We require information from authors about some types of materials, experimental systems and methods used in many studies. Here, indicate whether each material, system or method listed is relevant to your study. If you are not sure if a list item applies to your research, read the appropriate section before selecting a response.

Materials & experimental systems

n/a	Involved in the study
☒	☐ Antibodies
☒	☐ Eukaryotic cell lines
☒	☐ Palaeontology and archaeology
☒	☐ Animals and other organisms
☒	☐ Clinical data
☒	☐ Dual use research of concern

Methods

n/a	Involved in the study
☒	☐ ChIP-seq
☒	☐ Flow cytometry
☒	☐ MRI-based neuroimaging
